# Multi-omics analysis reveals the key factors involved in the severity of the Alzheimer’s disease

**DOI:** 10.1186/s13195-024-01578-6

**Published:** 2024-10-02

**Authors:** Lingqi Meng, Han Jin, Burak Yulug, Ozlem Altay, Xiangyu Li, Lutfu Hanoglu, Seyda Cankaya, Ebru Coskun, Ezgi Idil, Rahim Nogaylar, Ahmet Ozsimsek, Saeed Shoaie, Hasan Turkez, Jens Nielsen, Cheng Zhang, Jan Borén, Mathias Uhlén, Adil Mardinoglu

**Affiliations:** 1 Science for Life Laboratory, KTH - Royal Institute of Technology, Stockholm, Sweden; 2 Department of Neurology and Neuroscience, Faculty of Medicine, Alanya Alaaddin Keykubat University, Antalya, Turkey; 3 Department of Neurology, Faculty of Medicine, Istanbul Medipol University, Istanbul, Turkey; 4 Centre for Host-Microbiome Interaction’s, Faculty of Dentistry, Oral & Craniofacial Sciences, King’s College London, London, UK; 5 Department of Medical Biology, Faculty of Medicine, Atatürk University, Erzurum, Turkey; 6 Department of Biology and Biological Engineering, Chalmers University of Technology, Gothenburg, Sweden; 7 Department of Molecular and Clinical Medicine, University of Gothenburg and Sahlgrenska University Hospital, Gothenburg, Sweden

## Abstract

Alzheimer’s disease (AD) is a debilitating neurodegenerative disorder with a global impact, yet its pathogenesis remains poorly understood. While age, metabolic abnormalities, and accumulation of neurotoxic substances are potential risk factors for AD, their effects are confounded by other factors. To address this challenge, we first utilized multi-omics data from 87 well phenotyped AD patients and generated plasma proteomics and metabolomics data, as well as gut and saliva metagenomics data to investigate the molecular-level alterations accounting the host-microbiome interactions. Second, we analyzed individual omics data and identified the key parameters involved in the severity of the dementia in AD patients. Next, we employed Artificial Intelligence (AI) based models to predict AD severity based on the significantly altered features identified in each omics analysis. Based on our integrative analysis, we found the clinical relevance of plasma proteins, including SKAP1 and NEFL, plasma metabolites including homovanillate and glutamate, and *Paraprevotella clara* in gut microbiome in predicting the AD severity. Finally, we validated the predictive power of our AI based models by generating additional multi-omics data from the same group of AD patients by following up for 3 months. Hence, we observed that these results may have important implications for the development of potential diagnostic and therapeutic approaches for AD patients.

**Supplementary Information:**

The online version contains supplementary material available at 10.1186/s13195-024-01578-6.

## Introduction

Alzheimer’s disease (AD) is a multifaceted and progressive neurodegenerative disorder that poses a significant global health challenge [64]. AD is characterized by the accumulation of amyloid-beta plaques and neurofibrillary tangles in the brain, leading to cognitive impairment, memory loss, and functional decline [64]. While age is a well-established risk factor for AD, there is growing evidence to suggest that other factors, such as diabetes [60], accumulation of neurotoxic substances [43, 68], oxidative stress [15], and alteration of the microbiome [59], may also contribute to the development and progression of the AD. Despite extensive research efforts, the underlying mechanisms of AD pathogenesis remain poorly understood, partially due to the complexity of the disease and the presence of confounding factors.

To date, numerous studies have conducted single-omics analyses on AD, revealing significant differences in protein, metabolite, and microbial compositions between AD patients and healthy controls [10, 24, 54, 70]. With recent advancements in high-throughput sequencing, multi-omics approaches have emerged as powerful tools to address the complexity of AD [6]. These approaches integrate multiple types of biological data to provide a comprehensive view of molecular changes, revealing the underlying biological processes and interactions involved in disease pathogenesis. By leveraging these techniques, researchers can discover key biomarkers, identify molecular pathways, and reveal the host-microbe interactions that contribute to the development of the disease. For example, gut microbial signatures have been linked to mild cognitive impairment and it has been reported that it can modulate the metabolites associated with AD biomarkers [45]. Epigenomic analyses have shown dysregulation of transcription- and chromatin-gene feedback loops in AD [47]. These findings emphasize the potential of multi-omics approaches to identify novel biomarkers, reveal the disease mechanisms, discover therapeutic targets and eventually develop efficient treatment strategies for AD.

We have gained valuable insights on the development of complex diseases through our previous multi-omics integration analyses in various fields, including cardiovascular disease [23], acquired obesity [65], metabolic dysfunction-associated fatty liver disease [74], and COVID-19 [2]. Despite previous research establishing an understanding of the molecular and microbial differences between AD patients and healthy controls, multi-omics variations across different levels of disease severity remain poorly investigated. In this study, we conducted an unbiased and comprehensive multi-omics analysis of 87 AD patients, by utilizing plasma inflammatory proteomics and metabolomics as well as gut and saliva metagenomics data to investigate the global metabolic and inflammatory processes involved in the development of AD accounting the host and microbiome interactions. Our analysis revealed significant alterations in the plasma proteins and metabolites during the different stages of AD. By leveraging machine learning algorithms to integrate these multi-omics data, we identified key features of AD from multiple perspectives. Furthermore, we validated our findings in a follow-up cohort by recruiting the some of the individuals after three months to avoid the genetic differences between the individuals. The insights gained from our research shed light on the underlying molecular mechanisms driving the progression of AD.

## Result

### Clinical and demographic characteristics of AD patients stratified by ADAS-Cog scores

**Fig. 1:**
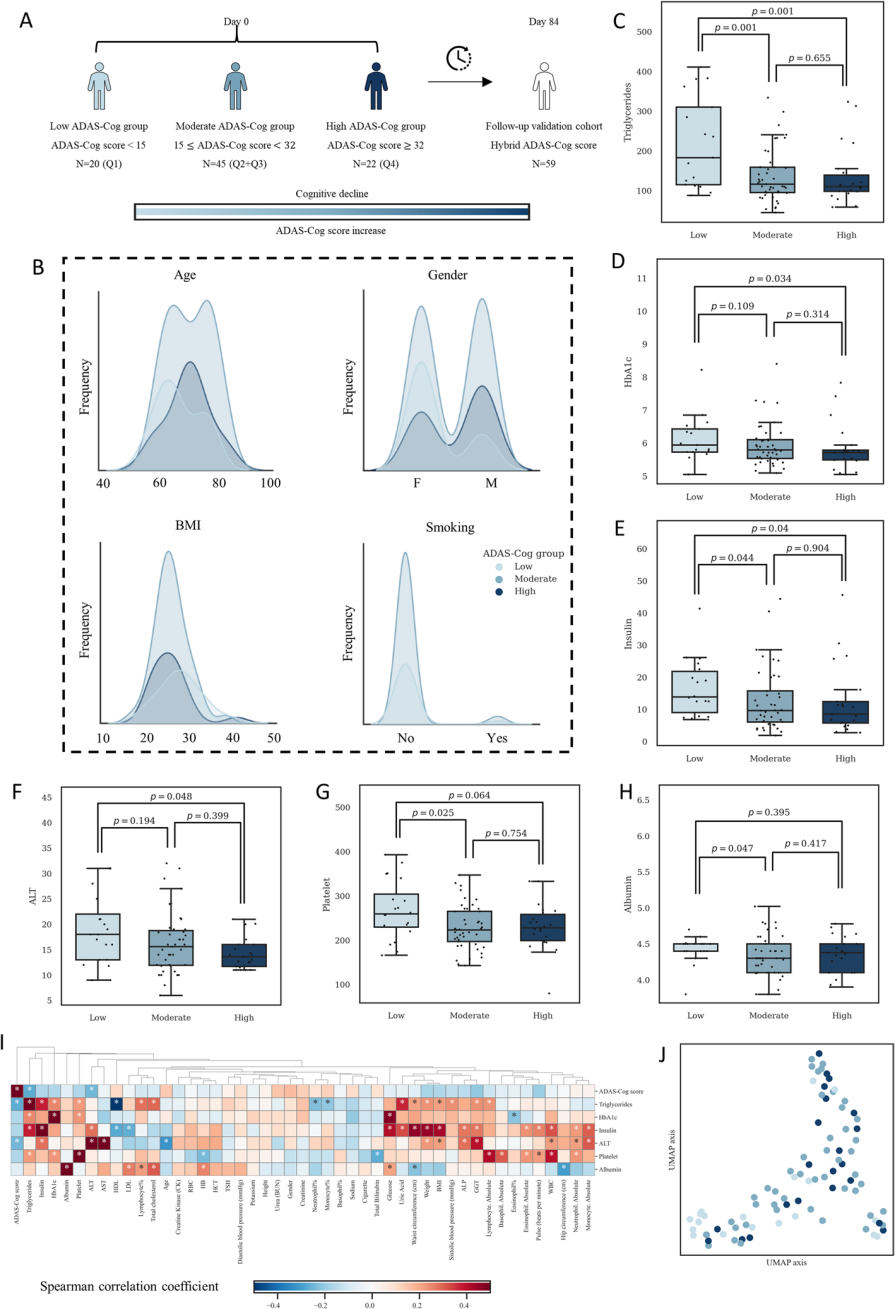
Overview of study design and analysis. **A** Classification of patients into low, moderate, and high ADAS-Cog groups based on quartiles. **B** Distribution of patient characteristics, including age, gender, BMI, and smoking habits. **C**-**H** Significant clinical parameters identified between ADAS-Cog groups. **I** Correlation analysis between significant and all clinical parameters. **J** UMAP visualization based on significant clinical parameters to depict inter-group differences

The study consisted of two phases: a baseline assessment on day 0 and a follow-up visit on day 84 (three months later). At the beginning of the study, 87 individuals diagnosed with AD were stratified into three groups based on their quartile scores on the Alzheimer’s Disease Assessment Scale—Cognitive Subscale (ADAS-Cog), which is a numerical value derived from the test that reflects the severity of cognitive impairment, with higher scores indicating greater impairment. ADAS-Cog score is the indicator of the cognitive function in AD patients. The low group consists of individuals with ADAS-Cog scores < 15 (*N* = 20), the moderate group consists of individuals with 15 ≤ ADAS-Cog scores < 32 (*N* = 45), and the high group consists of individuals with ADAS-Cog scores ≥ 32 (*N* = 22, Fig. 1A). Of the initial 87 patients, 59 completed the follow-up visit after 84 days (Fig. 1A). The first cohort with 87 patients has been employed as a finding cohort whereas the second cohort with 59 patients has been employed as a validation cohort.

The population had an average age of 70.39 years (standard deviation = 8.11, 95%CI = (68.65, 72.13)) and an average BMI of 28.77 (standard deviation = 5.33, 95%CI = (27.62, 29.92)). Of the participants, 41 were male and 46 were female, with 3 smokers and 84 non-smokers. A chi-squared test was performed to assess the distribution of demographic variables among the groups, and no significant differences were found regarding age, gender, body mass index, or smoking habits (*p* > 0.05, Fig. 1B, Supplementary Table 1).

The clinical data analysis revealed six significantly different variables among the groups stratified based on ADAS-Cog scores (Supplementary Table 1). Triglyceride levels were found to be decreased with the increasing ADAS-Cog scores, and the low group had a significantly higher median level than the moderate and high groups (*p* = 0.001, Fig. 1C). The median triglyceride level in the moderate group was higher than that in the high group, although this difference was not statistically significant. On the other hand, the low group had significantly higher HbA1c, insulin, and ALT levels compared to the high group (Fig. 1D-F). While differences between the low and moderate groups, as well as between the moderate and high groups, were not always significant, a trend towards decreasing median values of HbA1c, insulin, and ALT with increasing ADAS-Cog scores was observed. Platelet and albumin levels were also significantly higher in the low group compared to the moderate group (Fig. 1G and H). However, the difference between the moderate and high groups was not significant, with similar median values observed between the two groups.

A correlation analysis was performed to assess the relationship between ADAS-Cog scores and other variables (Fig. 1I). Results showed a significant negative correlation between ADAS-Cog scores and triglycerides and ALT levels (*p* < 0.05, Spearman’s rank test). Additionally, a positive correlation between ADAS-Cog scores and age was observed, although it was not statistically significant. A Uniform Manifold Approximation and Projection (UMAP) visualization was performed based on the six significant parameters, and it was observed that the patients from different groups were well mixed (Fig. 1J).

### Differential plasma inflammation proteins in AD patients with varying ADAS-Cog scores

We investigated differential plasma inflammation proteins in patients with AD patients, who had varying ADAS-Cog scores. Plasma protein concentrations were measured using normalized protein expression (NPX), an arbitrary unit on a log2 scale calculated from cycle threshold values.

**Fig. 2:**
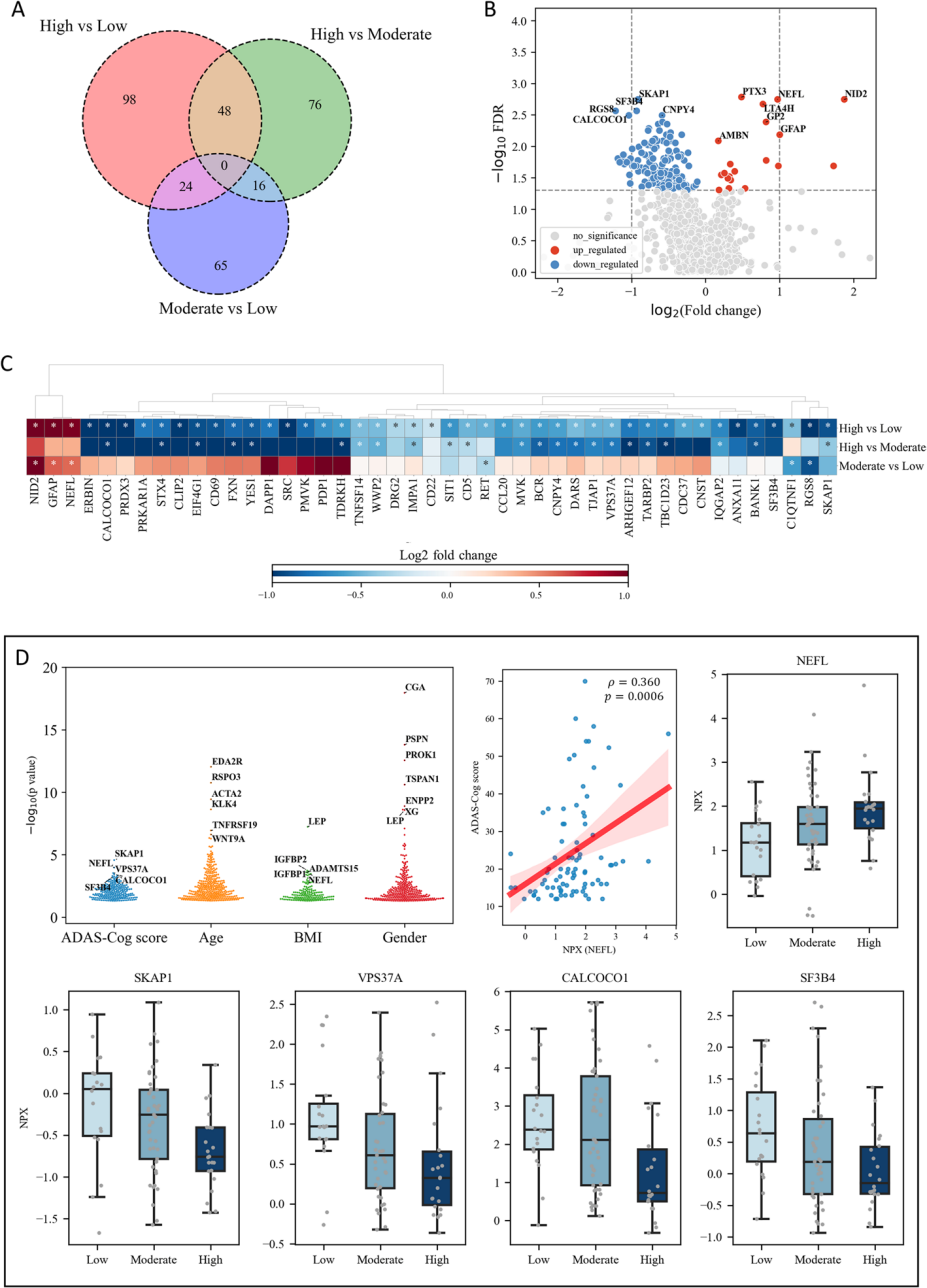
Analysis of plasma proteins in different patient groups. **A** Venn diagram depicting the overlap of significantly altered plasma proteins across all groups. **B** Volcano plot illustrating the plasma proteins that are significantly altered between high and low ADAS-Cog groups. **C** Heat map displaying the top 40 plasma proteins that are significantly altered between different patient groups. Asterisks indicate statistical significance with a threshold of *p* < 0.05. **D** Swarm plot depicting the correlation between plasma protein concentration and ADAS-Cog, age, BMI, and gender, as well as the top five significantly altered proteins

Our results revealed significant alterations in the plasma level of 170 plasma proteins between the high and low ADAS-Cog groups, with 150 downregulated and 20 upregulated proteins (Fig. 2A, Supplementary Table 2). We also identified 48 proteins that were significantly altered between the high and moderate ADAS-Cog groups, and 24 proteins that were significantly altered between the moderate and low ADAS-Cog groups (Fig. 2A, Supplementary Table 2). We identified the top 5 significantly downregulated proteins as SKAP1, SF3B4, CALCOCO1, RGS8, and CNPY4, while the top 5 upregulated proteins were PTX3, NEFL, NID2, LTA4H, and GP2 (Fig. 2B). We investigated the log2 fold change of NPX between the high versus low, high versus moderate, and moderate versus low ADAS-Cog groups (Fig. 2C). Our analysis revealed significant increases in the level of NEFL, GFAP, and NID2 in plasma concentrations in the high and moderate ADAS-Cog groups compared to the low group. SKAP1, SF3B4, CALCOCO1, and VPS37A concentrations were found to be lower in the high ADAS-Cog group compared to the moderate and low groups. Additionally, SKAP1 and SF3B4 concentrations were lower in the moderate group than in the low group, although the difference was not statistically significant. We also conducted a Spearman correlation analysis to investigate the relationship between plasma proteomics NPX and ADAS-Cog scores, age, BMI, and gender. Of the correlated proteins, SKAP1, NEFL, VPS37A, CALCOCO1, and SF3B4 showed the strongest correlation with ADAS-Cog scores, and their detailed comparison across the three ADAS-Cog groups is presented in Fig. 2D (Supplementary Table 2). The correlation plots for these proteins was presented in Fig. 2D and Supplementary Figure 2. We found that LEP is top protein that is significantly correlated with BMI, with higher levels in females (Supplementary Table 2). It is well-known that leptin levels in the blood are positively correlated with adipose tissue mass [34], and it could serve as a positive control of our analysis. Therefore, our analysis indicates that SKAP1, NEFL, VPS37A, CALCOCO1 and SF3B4 could play important roles in the development of AD.

We investigated the associations between six clinical variables and the top 40 plasma levels of inflammation-related proteins in AD patients (Supplementary Figure 1). Two main clusters of proteins were identified. NEFL and GFAP were found to be negatively correlated with triglycerides, insulin, and ALT, but positively correlated with ADAS-Cog scores. SKAP1, VPS37A, CALCOCO1, and SF3B4, on the other hand, were positively correlated with triglycerides, HbA1c, and platelet count. Prior research has demonstrated that SKAP1 (alias SKAP55) deficient platelets exhibit impaired activation and aggregation in response to various stimuli [31]. SKAP1 has also been shown to interact with other proteins involved in platelet function, including the integrin αIIbβ3 [30]. We also observed that plasma level of RET was correlated with all the significant clinical parameters examined in this study.

### Differential plasma metabolites in AD patients with varying ADAS-Cog scores

We performed an untargeted metabolomics analysis on the plasma samples obtained from 87 AD patients, measured the concentrations of 1142 metabolites and identified the differential metabolites between the groups. After excluding metabolites with missing values in over 50% of the samples, 982 metabolites were included in subsequent analyses. The aim of this analysis was to identify the key metabolites associated with underlying molecular mechanisms related to ADAS-Cog scores and cognitive function in AD patients.

**Fig. 3:**
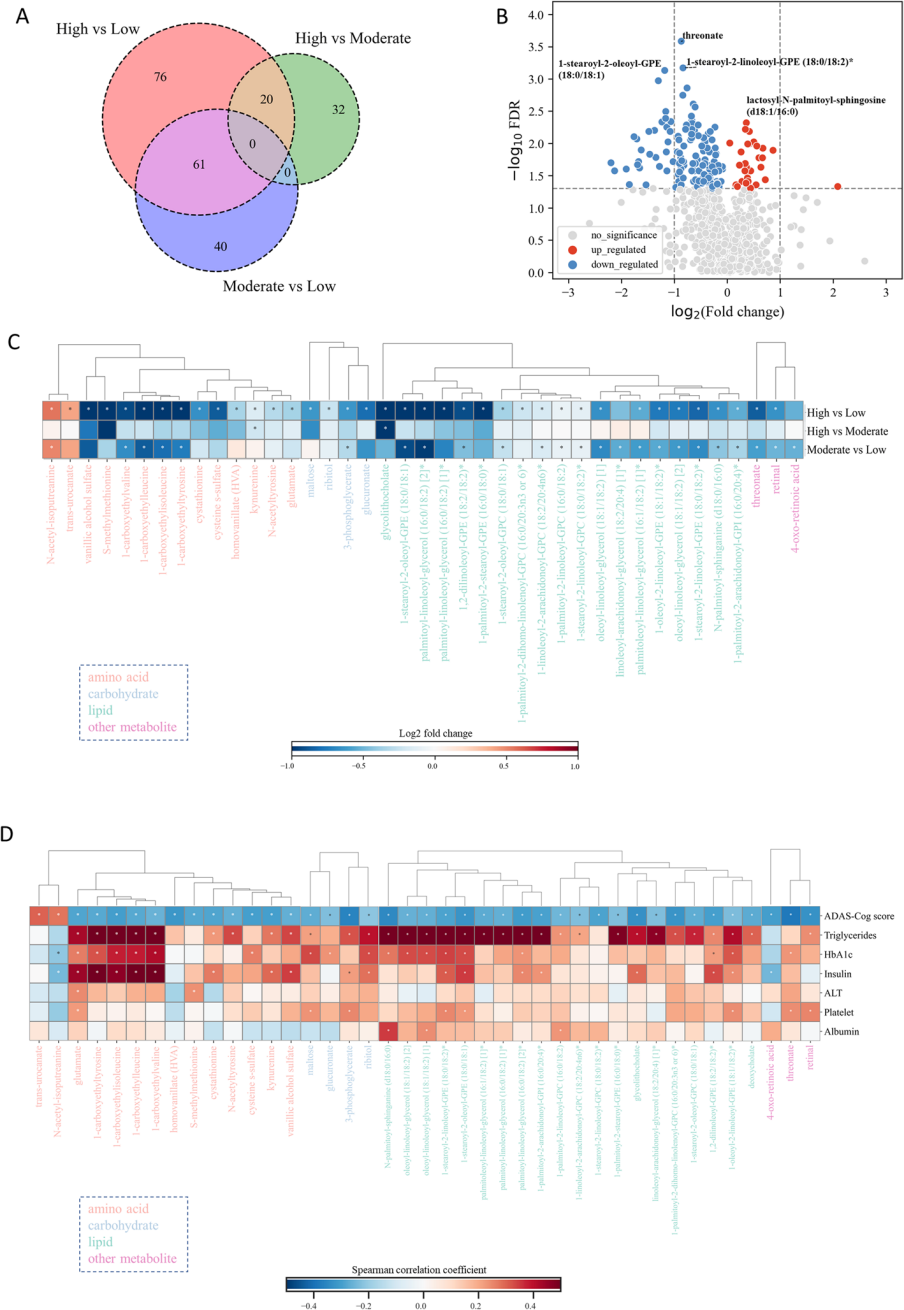
Analysis of plasma metabolites in different ADAS-Cog groups. **A** Venn diagram depicting the overlap of significantly altered plasma metabolites across all groups. **B** Volcano plot illustrating the plasma metabolites that are significantly altered between high and low ADAS-Cog groups. **C** Heat map displaying the top 40 plasma metabolites that are significantly altered between different patient groups. Asterisks indicate statistical significance with a threshold of *p* < 0.05. **D** Correlation analysis between significant clinical parameters and the top 40 plasma metabolites

We conducted group pairwise analyses to identify metabolites that were significantly different between the ADAS-Cog groups. We found that 157 metabolites were significantly different between high and low ADAS-Cog groups, while 20 metabolites were significantly different between high and moderate ADAS-Cog groups, and 61 metabolites were significantly different between moderate and low ADAS-Cog groups (Fig. 3A, Supplementary Table 3). Notably, we observed significant downregulation of threonate, phosphatidylethanolamines (PEs; 1-stearoyl-2-linoleoyl-GPE (18:0/18:2)* and 1-stearoyl-2-oleoyl-GPE (18:0/18:1)), and diacylglycerols (DAGs; palmitoyl-linoleoyl-glycerol (16:0/18:2) [2]* and oleoyl-linoleoyl-glycerol (18:1/18:2) [2]), while significantly upregulation of plasmalogens (1-(1-enyl-palmitoyl)-2-palmitoyl-GPC (P-16:0/16:0)* and 1-(1-enyl-palmitoyl)-2-palmitoleoyl-GPC (P-16:0/16:1)*), lactosyl-N-palmitoyl-sphingosine, trans-urocanate, and 2-ethylphenylsulfate in the different ADAS-Cog groups (Fig. 3B, Supplementary Table 3).

We classified the tested plasma metabolites into amino acids, carbohydrates, lipids, xenobiotics, and other categories, as detailed in Supplementary Table 3. We evaluated the Spearman’s correlation between plasma metabolites and ADAS-Cog scores, and observed changes in the top 40 metabolites across the different ADAS-Cog groups (Fig. 3C). Compared to the low ADAS-Cog group, most metabolites were downregulated in the moderate and high groups. Notably, PEs (1-stearoyl-2-oleoyl-GPE (18:0/18:1), 1,2-dilinoleoyl-GPE (18:2/18:2)*, 1-stearoyl-2-linoleoyl-GPE (18:0/18:2)*, 1-oleoyl-2-linoleoyl-GPE (18:1/18:2)*, and 1-palmitoyl-2-stearoyl-GPE (16:0/18:0)*) and phosphatidylcholines (PCs; 1-palmitoyl-2-dihomo-linolenoyl-GPC (16:0/20:3n3 or 6)*, 1-stearoyl-2-linoleoyl-GPC (18:0/18:2)*, 1-stearoyl-2-oleoyl-GPC (18:0/18:1), 1-palmitoyl-2-linoleoyl-GPC (16:0/18:2), and 1-linoleoyl-2-arachidonoyl-GPC (18:2/20:4n6)*) were significantly downregulated in the moderate and high groups, while glutamate, kynurenine, homovanillate (HVA), vanillic alcohol sulfate (VAS), glycolithocholate, and threonate were downregulated in the high group compared to the low group. We present a complete groupwise differential analysis of amino acids and lipids in Supplementary Figure 3. Our analysis also revealed a significant downregulation of amino acid-related pathways, including tyrosine metabolism, glutamate metabolism, branched-chain amino acids (BCAAs), and lysine metabolism, as ADAS-Cog scores increased (Supplementary Figure 3A, Supplementary Table 3). Nearly all tested lipids, including PCs and PEs, were downregulated as ADAS-Cog scores increased, except for some plasmalogens (1-(1-enyl-palmitoyl)-2-palmitoyl-GPC (P-16:0/16:0), 1-(1-enyl-palmitoyl)-2-palmitoleoyl-GPC (P-16:0/16:1), and 1-(1-enyl-palmitoyl)-2-oleoyl-GPC (P-16:0/18:1)*, Supplementary Figure 3B, Supplementary Table 3).

We investigated the potential associations between six clinical variables and the top 40 plasma levels of metabolites in patients with AD (Fig. 3D). Our results demonstrate that the majority of the identified metabolites were positively correlated with the clinical variables examined. Amino acids, including glutamate, 1-carboxyethylisoleucine, 1-carboxyethylleucine, 1-carboxyethyltyrosine, and 1-carboxyethylvaline, as well as PEs, such as 1-stearoyl-2-oleoyl-GPE (18:0/18:1), 1-stearoyl-2-linoleoyl-GPE (18:0/18:2), 1-oleoyl-2-linoleoyl-GPE (18:1/18:2), and 1,2-dilinoleoyl-GPE (18:2/18:2), and carbohydrate (ribitol) were found to be strongly positively correlated with triglycerides, HbA1c, and insulin. Similarly, PCs, including 1-palmitoyl-2-dihomo-linolenoyl-GPC (16:0/20:3n3 or 6)*, 1-stearoyl-2-oleoyl-GPC (18:0/18:1), and 1-palmitoyl-2-linoleoyl-GPC (16:0/18:2), as well as tyrosine and kynurenine, were found to be strongly positively correlated with triglycerides. Moreover, N-palmitoyl-sphinganine (d18:0/16:0) was strongly positively correlated with triglycerides, HbA1c, and albumin. Of note, although homovanillate and 1-stearoyl-2-linoleoyl-GPC (18:0/18:2)* were not significantly correlated with the six clinical parameters, they were also found to be strongly significantly correlated with ADAS-Cog scores. As a positive control of our analysis, elevated levels of BCAAs, such as 1-carboxyethylisoleucine and 1-carboxyethylleucine, have been linked to insulin resistance, which may contribute to increased blood glucose levels and the risk of type 2 diabetes [42]. Additionally, the dihydroceramide N-palmitoyl-sphinganine (d18:0/16:0) has been reported to be involved in the synthesis and secretion of triglyceride-rich very-low-density lipoprotein, which is predictive of type 2 diabetes and related metabolic dysfunctions [16].

### The dysbiosis of the gut and saliva microbiome in AD patients

We investigated the potential role of the gut and saliva microbiome in AD based on shot gun metagenomics analysis, analyzed the microbial composition and assessed the dysbiosis in the gut and saliva microbiomes of subjects in response to varying ADAS-Cog scores. Our analysis focused on the alternation between high versus low ADAS-Cog groups. Additional analyses comparing moderate versus low and high versus moderate groups can be found in Supplementary Figure 4A–D (Supplementary Table 4 and 5).

**Fig. 4:**
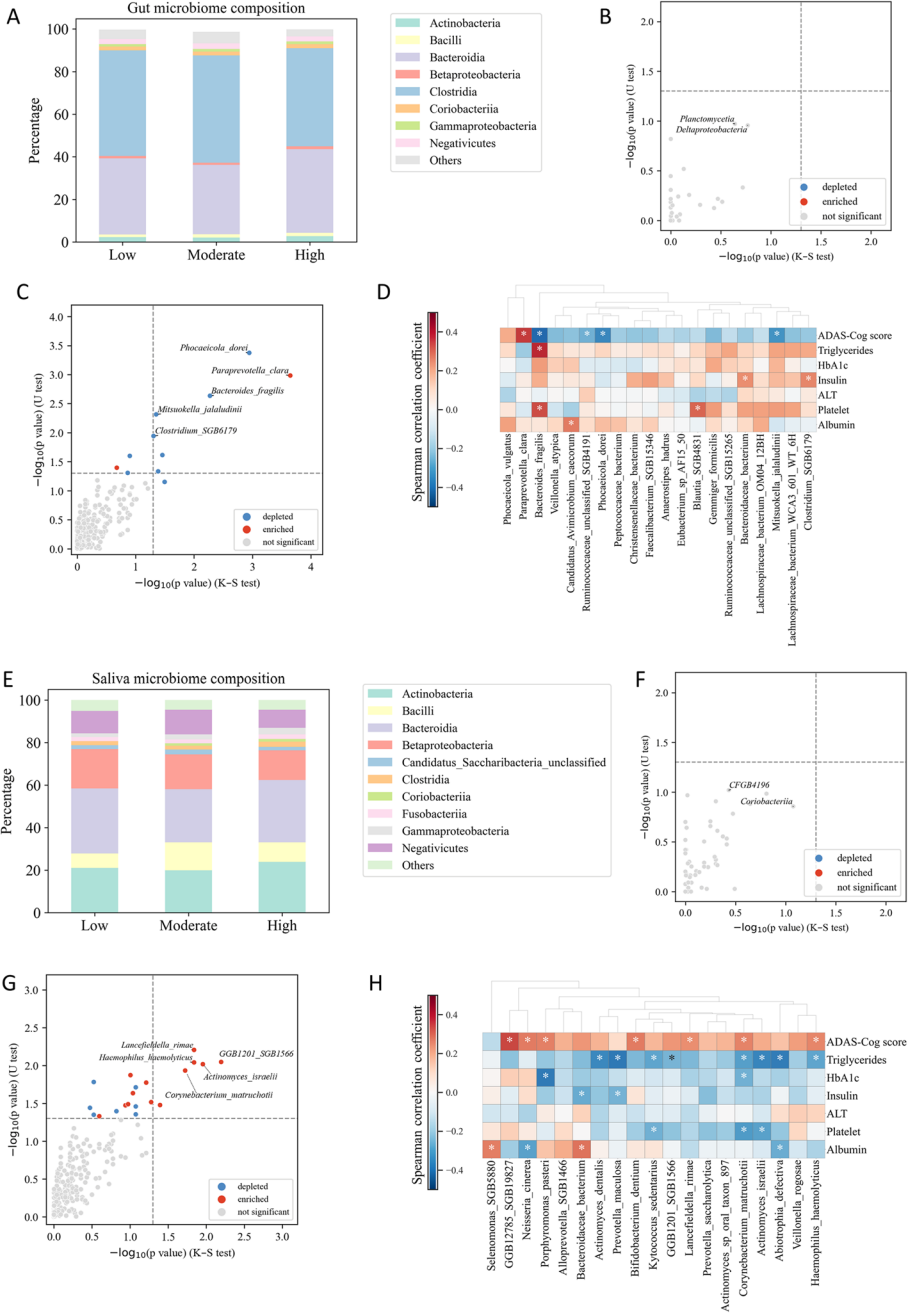
Analysis of gut and saliva microbiome composition and dysbiosis in different patient groups. **A** Relative abundance of taxa at the class level in the low, moderate, and high groups. Taxa with relative abundance < 1% are categorized as “others”. **B** Comparison of altered classes between high and low groups using the Kolmogorov–Smirnov and Mann–Whitney U tests. A class is considered significantly altered if it passes the dotted horizontal or vertical line. **C** Significant gut species that differ between high and low groups. **D** Correlation between top 20 gut microbiomes and significant clinical parameters. **E** Relative abundance of taxa at the class level in the low, moderate, and high groups for saliva microbiome. **F** Comparison of altered classes between high and low groups for saliva microbiome. **G** Significant saliva species that differ between high and low groups. **H** Correlation between top 20 saliva microbiomes and significant clinical parameters

To visualize the composition of the gut microbiome in different groups, we classified the microbial species at the class level of taxonomy. *Bacteroidia* (*Bacteroidetes*) and *Clostridia* (*Firmicutes*) accounted for about 80% of the total classes’ abundance (Fig. 4A). We used the Kolmogorov–Smirnov test to identify classes with significant distribution across groups and the Mann–Whitney U test to identify statistically different classes. The gut microbiome class composition was similar across the different ADAS-Cog groups, as indicated by the lack of significant differences (Fig. 4B). We observed that *Deltaproteobacteria* had a relatively small *p*-value (Fig. 4B). Previous research has suggested that higher levels of certain types of *Proteobacteria*, such as *Escherichia coli* [39] and *Helicobacter pylori* [35], may increase the risk of AD.

At the species level, we focused only on gut microbiomes with a prevalence of at least 20% in our cohort. We observed that 11 gut microbiomes were significantly altered between high and low ADAS-Cog groups, belonging either to *Firmicutes* or *Bacteroidetes*. *Firmicutes* species, including *Mitsuokella jalaludinii* and *Clostridium SGB6179*, were downregulated in the high ADAS-Cog group (Fig. 4C). *Bacteroidetes* species, including *Phocaeicola dorei* and *Bacteroides fragilis*, were significantly decreased, while *Paraprevotella clara* was significantly increased in the high ADAS-Cog group (Fig. 4C).

We conducted an investigation into the potential associations between six clinical parameters and the top 20 gut microbiomes in patients with AD (Fig. 4D). Our results revealed that certain gut microbiomes including *Phocaeicola dorei*, *Bacteroides fragilis*, and *Mitsuokella jalaludinii* were significantly and negatively correlated with ADAS-Cog scores. Conversely, *Paraprevotella clara* was significantly and positively correlated with ADAS-Cog scores. Furthermore, *Bacteroides fragilis* was significantly and positively correlated with triglycerides and platelet, while *Clostridium SGB6179* was significantly and positively correlated with insulin levels.

The analysis of the saliva microbiome identified *Actinobacteria*, *Bacilli* (*Firmicutes*), *Bacteroidia* (*Bacteroidetes*), *Betaproteobacteria*, and *Negativicutes* (*Firmicutes*) as the dominant classes, comprising about 80% of the total class abundance (Fig. 4E). The microbiome class composition was similar across the different ADAS-Cog groups, as indicated by the lack of significant differences (Fig. 4F). However, Coriobacteriia, which is present in both gut and saliva microbiomes, had the smallest relative *p*-value (Fig. 4F), although its relationship with AD is yet to be elucidated.

At the species level, we identified significant alterations in 20 different species, including eight *Actinobacteria*, seven *Bacteroidetes*, one *Candidatus Saccharibacteria*, three *Firmicutes*, and two *Proteobacteria* (Fig. 4G). Notably, *Actinobacteria* species such as *Actinomyces israelii*, *Corynebacterium matruchotii*, *Lancefieldella rimae*, and others were found to be upregulated in the high ADAS-Cog group, while most *Bacteroidetes* species, including *Porphyromonas pasteri* and *GGB1201 SGB1566*, were also upregulated. Additionally, the *Proteobacteria* species *Haemophilus haemolyticus* was found to be upregulated. Interestingly, previous research has shown that *Porphyromonas gingivalis*, a bacterium closely related to *Porphyromonas pasteri*, and its toxic proteases have been detected in the brains of individuals with AD, and the levels of these bacteria and proteases were positively correlated with the severity of AD pathology [22].

Correlation analysis between the six clinical parameters and top 20 saliva microbiomes revealed significant associations with cognitive function and lipid metabolism (Fig. 4H). Specifically, the abundance of two *Actinobacteria* species, *Corynebacterium matruchotii* and *Cryptobacterium curtum*, as well as four *Bacteroidetes* species (*Alloprevotella SGB1466*, *Bacteroidaceae bacterium*, *GGB1201 SGB1566*, and *Porphyromonas pasteri*) were found to be positively correlated with ADAS-Cog scores. Furthermore, one *Firmicutes* species, *Veillonella rogosae*, was also found to be positively correlated with ADAS-Cog scores. In contrast, we observed a significant negative correlation between the abundance of several Actinobacteria species (*Actinomyces dentalis*, *Actinomyces israelii*, *Corynebacterium matruchotii*, and *Kytococcus sedentarius*) and triglyceride levels.

The analysis presented utilized MetaPhlAn4 as the primary tool [13]. Furthermore, we conducted additional analysis using the previous pipeline, MetaPhlAn3, which yielded comparable outcomes, as visually demonstrated in Supplementary Figure 4E and F (Supplementary Table 4 and 5).

### Association among different omics in AD patients

**Fig. 5:**
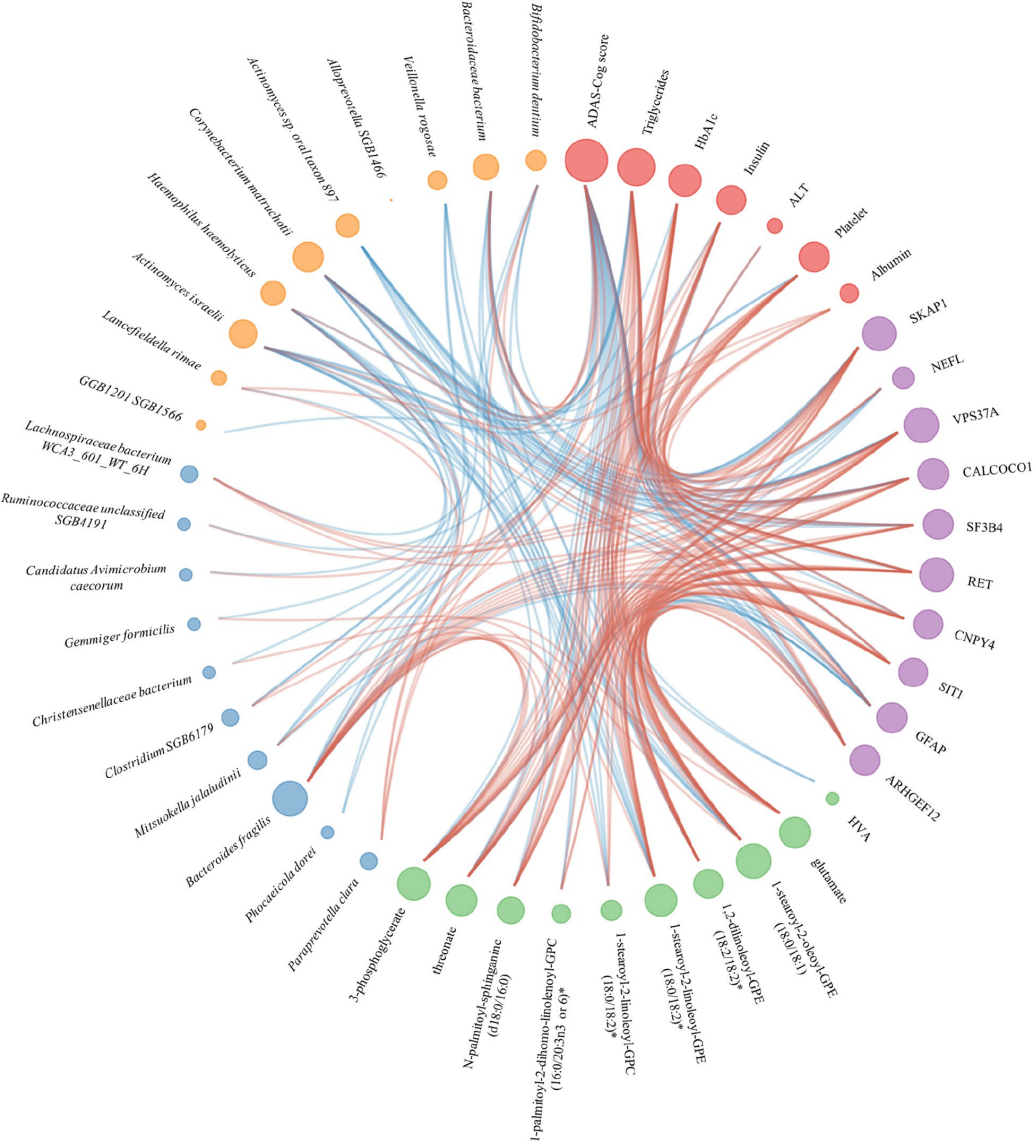
Integration of phenomics, metabolomics, proteomics, and gut/saliva metagenomics using a network approach. Only significant clinical parameters were included, while the other omics data included the top 10 features. Node size is proportional to its degree, and edges are colored red/blue to indicate positive/negative correlation between two nodes

We employed a network-based approach to capture the interplay between various omics data (Fig. 5). Our network comprised 47 nodes, which represented the key clinical and omics features associated with AD, including the ADAS-Cog score, six significant clinical features from our clinical analysis, and the top 10 features from proteomics, metabolomics, gut metagenomics and saliva metagenomics. To establish the functional relationships among these features, we computed Spearman’s correlation coefficients and visualized them as red/blue edges between the corresponding nodes. The size of each node in the network was proportional to its degree. For readers who are interested in the entire network, we included a plot of interomics correlation in Supplementary Figure 5.

We investigated the associations between the top 40 significant plasma levels of metabolites and the top 10 significant plasma inflammatory proteins in AD patients (Supplementary Figure 5A). Our results suggested that elevated levels of amino acids (glutamate, kynurenine, cysteine s-sulfate, and cystathionine), several BCAAs (1-carboxyethylisoleucine, 1-carboxyethylleucine, 1-carboxyethyltyrosine, and 1-carboxyethylvaline), carbohydrates (3-phosphoglycerate and maltose), and PEs (1,2-dilinoleoyl-GPE (18:2/18:2)* and 1-oleoyl-2-linoleoyl-GPE (18:1/18:2)*) were significantly positively correlated with VPS37A, CALCOCO1, and SKAP1. In particular, SKAP1 is also significantly correlated with PCs (1-palmitoyl-2-dihomo-linolenoyl-GPC (16:0/20:3n3 or 6)* and 1-stearoyl-2-linoleoyl-GPC (18:0/18:2)*), N-palmitoyl-sphinganine (d18:0/16:0), and tyrosine. Amino acids (gutamate, cysteine s-sulfate, and cystathionine) and 1,2-dilinoleoyl-GPE (18:2/18:2)* are significantly negatively correlated with NEFL and GFAP. HVA is only significantly positively correlated with CNPY4.

We investigated the potential associations between the gut/saliva microbiome and top 10 significant plasma inflammatory proteins. In the gut microbiome (Supplementary Figure 5B), *Bacteroides fragilis* showed significant positive correlations with SKAP1, SF3B4, and VPS37A, and significant negative correlations with NEFL and GFAP. *Mitsuokella jalaludinii* was significantly positively correlated with VPS37A, and significantly negatively correlated with GFAP. In the saliva microbiome (Supplementary Figure 5C), four *Actinobacteria* species (*Actinomyces dentalis*, *Actinomyces israelii*, *Actinomyces sp_oral_taxon_897*, and *Corynebacterium matruchotii*) and two *Bacteroidetes* species (*Bacteroidaceae bacterium* and *Porphyromonas pasteri*) were significantly negatively correlated with VPS37A, CALCOCO1, SF3B4, and ARHGEF12. SKAP1 was significantly negatively correlated with *Actinomyces dentalis*, *Actinomyces sp_oral_taxon_897*, *Corynebacterium matruchotii*, and *Porphyromonas pasteri*. NEFL and GFAP were significantly positively correlated with *Actinomyces dentalis*, *Actinomyces sp_oral_taxon_897*, *Bacteroidaceae bacterium*, and *Oribacterium sp_oral_taxon_078*.

We investigated the potential associations between plasma metabolites and the gut and saliva microbiomes. We identified significant correlations between the top 10 significant plasma metabolites and the top 20 microbial species in the gut and saliva microbiomes. In the gut microbiome (Supplementary Figure 5D), *Bacteroides fragilis* was significantly positively correlated with N-palmitoyl-sphinganine (d18:0/16:0) and PEs (1-stearoyl-2-oleoyl-GPE (18:0/18:1) and 1-stearoyl-2-linoleoyl-GPE (18:0/18:2)). *Mitsuokella jalaludinii* and *Clostridium SGB6179* were significantly positively correlated with 3-phosphoglycerate. In the saliva microbiome (Supplementary Figure 5E), glutamate was significantly negatively correlated with *Abiotrophia defectiva*. Additionally, HVA was significantly negatively correlated with *Veillonella rogosae*. 1-stearoyl-2-linoleoyl-GPC (18:0/18:2) was significantly negatively correlated with *Veillonella rogosae* and *Haemophilus haemolyticus*, while PEs (1-stearoyl-2-oleoyl-GPE (18:0/18:1) and 1-stearoyl-2-linoleoyl-GPE (18:0/18:2)*) were significantly negatively correlated with *Haemophilus haemolyticus*, *Abiotrophia defectiva*, *Actinomyces israelii*, and *Actinomyces sp_oral_taxon_897*.

Our study investigated the associations between the top 20 gut and saliva microbiomes, as shown in Supplementary Figure 5F. Our results reveal several significant correlations between different bacterial species. *Haemophilus haemolyticus* exhibited a significant positive correlation with *Paraprevotella clara* and a significant negative correlation with *Phocaeicola dorei. Bacteroides fragilis* and *Mitsuokella jalaludinii* were significantly negatively correlated with *Abiotrophia defective*, *Corynebacterium matruchotii*, *Kytococcus sedentarius*, and *Porphyromonas pasteri*.

### Prediction of ADAS-Cog scores based on multi-omics factor analysis

**Fig. 6:**
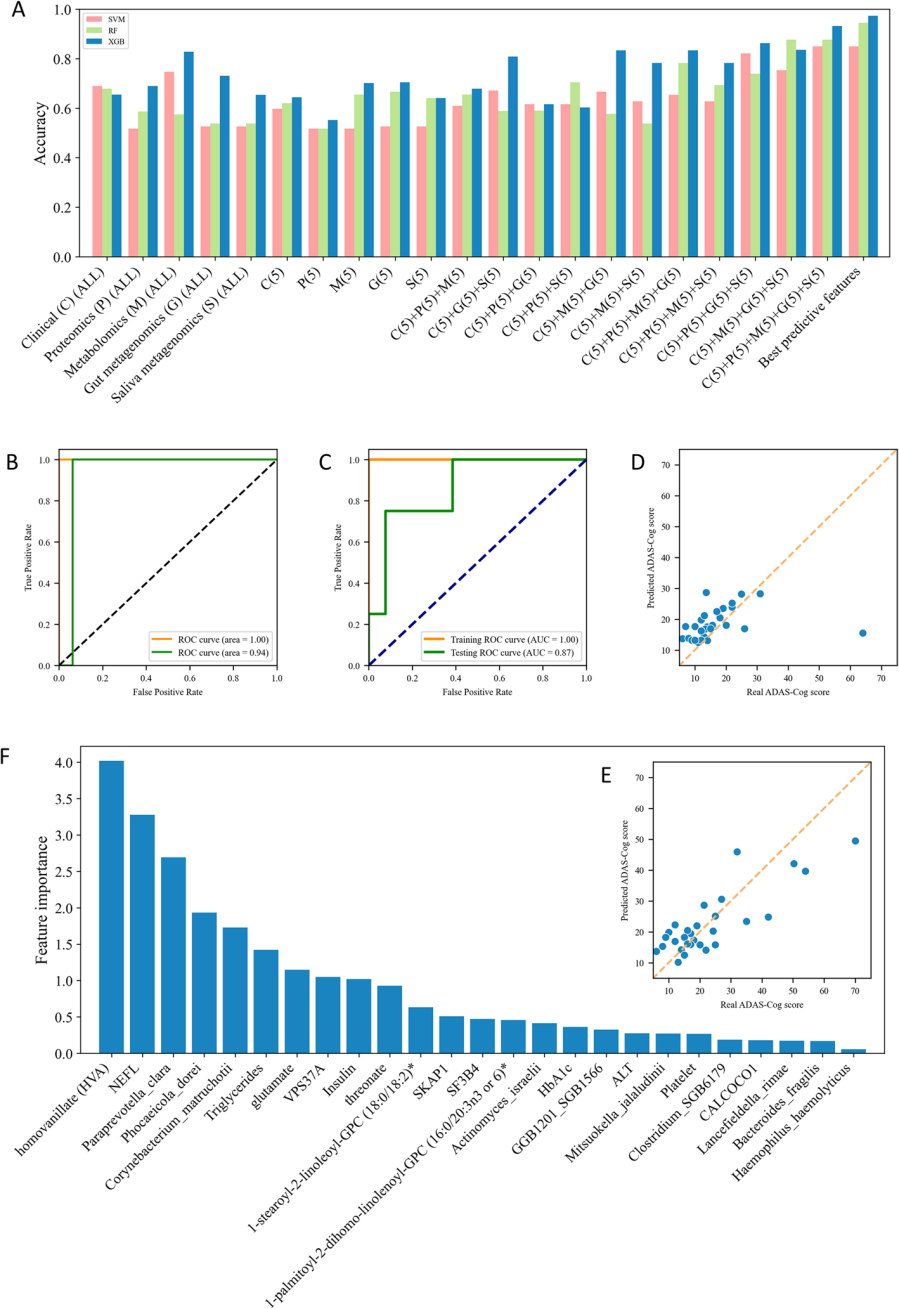
Application of Machine Learning Techniques in Multi-Omics Integration. **A** Accuracy of SVM, RF, and XGBoost algorithms in classifying AD patients. **B** ROC curve for the classification of AD patients without imputation, applied on the training (day 0) and testing (day 84) datasets. **C** ROC curve for classification of AD patients with imputation, with the training (80%) and testing (20%) datasets. **D** The performance of the XGBoost regression model applied to the testing dataset (day 84). **E** The performance of the XGBoost regression model on the imputed testing dataset (20%). **F** Feature importance as identified on the imputed testing (20%) dataset using the XGBoost algorithm

We conducted a comprehensive analysis of multi-omics datasets to investigate the distinguishing features among subjects with varying ADAS-Cog groups in our cohort of 87 AD patients. The datasets included phenomics, metabolomics, proteomics, and gut/saliva metagenomics. To analyze the individual data points from each omics dataset, we employed support vector machine (SVM), random forest (RF), and XGBoost algorithms, as depicted in Fig. 6A. To prevent overfitting, we carefully tuned hyperparameters, such as penalized regularization factors, maximum tree depth, and learning rate, using fivefold cross-validation.

Our findings confirmed that metabolomics and gut metagenomics exhibited high accuracy in single-omics classification (Fig. 6A). Moreover, in the case of multi-omics classification, all three machine learning algorithms generally outperformed the predictions based on single-omics data. Notably, XGBoost consistently demonstrated superior performance compared to SVM and RF in most prediction models. Specifically, when we selected the top 5 features from each omics dataset to classify the ADAS-Cog group, XGBoost achieved the highest accuracy of 0.931 (Fig. 6A). Furthermore, we performed classification using the top 25 features identified through ANOVA F-value analysis across all omics data, resulting in an accuracy of 0.973 for XGBoost (Fig. 6A).

We placed emphasis on the performance of the XGBoost classifier, which made use of the top 5 features from each omics dataset in distinguishing between the low and high ADAS-Cog groups. As outlined in Fig. 1, we previously conducted a randomized, double-blind, placebo-controlled phase-II clinical trial, which sought to explore the impact of combined metabolic activators on Alzheimer’s disease (AD) patients [73]. This study unfolded over a 12-week timeframe, with a final count of 59 AD patients attending the follow-up visit. Of these, 28 patients were able to provide a complete set of multi-omics data by the 84th day. With a focus on assessing the broader applicability of our XGBoost model on separate testing cohorts, the baseline cohort was utilized to train the classifier, while its performance was subsequently evaluated on the day 84 cohort. The Area Under the Receiver Operating Characteristic (ROC) Curve (AUC) for the training baseline dataset achieved a score of 1.00, an outcome indicative of superior discriminatory power (Fig. 6B). When tested on the day validation cohort, the AUC maintained a commendable score of 0.94. This performance attests to the robustness of the classifier and its capacity to generalize to unfamiliar data (Fig. 6B). We implemented the k-nearest neighbors (KNN) imputation algorithm to account for the missing values within the follow-up cohort. Subsequently, the data from both day 0 and day 84 were integrated. This combined cohort was then subjected to a random split, with 80% serving as the training set and the remaining 20% as the test set. The outcome revealed an AUC of 1.00 for the training baseline dataset, reaffirming the model’s impressive discriminatory prowess. Moreover, the AUC of the test dataset was also notably high at 0.87 (Fig. 6C).

In an extended evaluation of our model’s predictive performance, we visually represented the regression results juxtaposed against the actual ADAS-Cog scores for each patient who provided a complete multi-omics data set on day 84 (Fig. 6D). The diagonal dotted line in the figure demarcates the ideal prediction outcome. Our regression model demonstrated a commendable low bias for ADAS-Cog scores that did not exceed 35. Additionally, we further scrutinized the predictive performance of the XGBoost model on the imputed testing dataset. The findings from this exercise revealed a notable degree of predictability across all ADAS-Cog groups. Specifically, the model maintained a relatively low Mean Absolute Error (MAE) of 6.20, further bolstering the model’s credibility in its ability to reliably forecast cognitive function across varying ADAS-Cog scores.

We employed the SHapley Additive exPlanations (SHAP) feature importance algorithm, as outlined by the reference [41], to discern the most influential parameters within the XGBoost model on the imputed testing dataset (Fig. 6F). The analysis identified the top ten predictive features, which were primarily proteins (NEFL and VPS37A), metabolites (HVA, glutamate, and threonate), and gut/saliva microbiota (Paraprevotella clara, Corynebacterium matruchotii, and Phocaeicola dorei). It is noteworthy that the majority of these identified markers have been previously associated with AD in various studies, thus reinforcing their potential as viable biomarkers for AD. Subsequently, we applied the same analytical process to the validation cohort that was devoid of any imputation. The findings from this assessment were analogous to those of the imputed testing dataset, corroborating the original results and further cementing the identified parameters as key influential features (Supplementary Figure 6A).

We utilized the Multi-Omics Factor Analysis (MOFA +) framework, a robust matrix factorization technique, to integrate and analyze multiple omics datasets to identify the common and distinct biological factors that underlie complex biological processes [5]. Our analysis utilized 10 factors, and we found that clinical, proteomics, and metabolomics data were the most critical factors in distinguishing the patients (Supplementary Figure 6B). To identify the crucial features that drove the integration analysis, we focused on the first five factors and visualized their feature importance in each omics dataset. Our results showed that triglycerides, lymphocyte, and platelet counts contributed most to the integration analysis (Supplementary Figure= 6C). Several proteins, such as CALCOCO1, ARHGEF12, VPS37A, GFAP, and SF3B4, had a higher weight than other proteins (Supplementary Figure 6D). Amino acids (1-carboxyethylleucine, 1-carboxyethyltyrosine, and vanillic alcohol sulfate) were the most important metabolites. Additionally, PEs (1-stearoyl-2-oleoyl-GPE (18:0/18:1), 1,2-dilinoleoyl-GPE (18:2/18:2), 1-stearoyl-2-linoleoyl-GPE (18:0/18:2), and 1-oleoyl-2-linoleoyl-GPE (18:1/18:2)*) had greater importance than other metabolites (Supplementary Figure 6E).

## Discussion

**Fig. 7:**
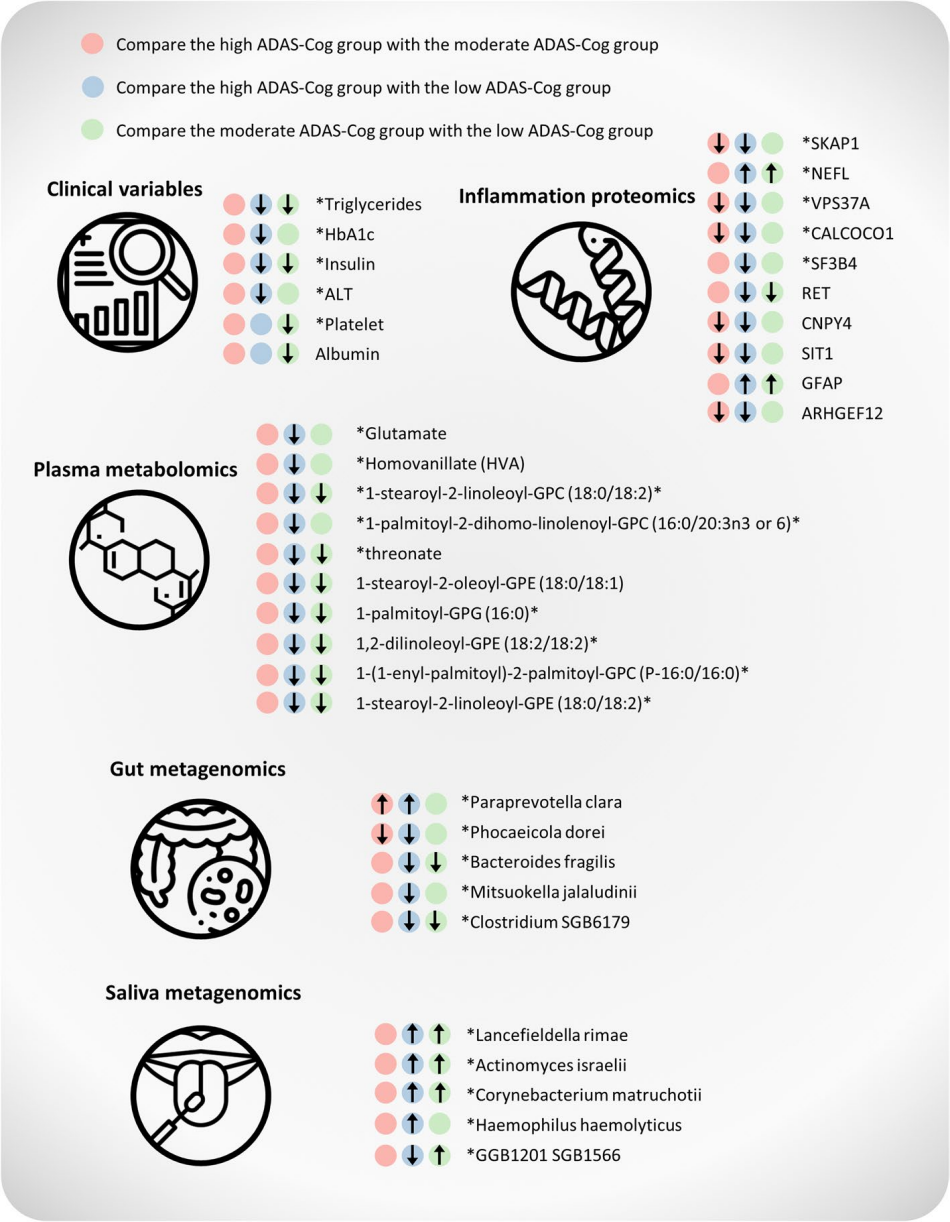
Key changes in phenomics, plasma proteomics, plasma metabolomics, gut and saliva microbiome in the context of ADAS-Cog score prediction. Asterisks indicate the parameters that were used in the random forest regression model

In this study, we presented the results of a comprehensive multi-omics analysis, which included phenomics, proteomics, metabolomics, and gut/saliva metagenomics, conducted on 87 AD patients. The most significant parameters associated with AD are summarized in Fig. 7. Notably, we observed that most parameters exhibited alterations in the same direction.

The traditional methods for diagnosing cerebral AD pathophysiology involve imaging and CSF measurements of Aβ, which have been considered gold standards [14]. However, these methods have several limitations that can make them difficult to use in routine clinical assessments of cognitive complaints. For instance, positron emission tomography imaging is expensive, and access to the ligand is limited, which may limit its widespread use until therapies become available. Moreover, brain studies are more invasive, and some studies are conducted posthumously, which can limit their utility in clinical settings [47]. CSF collection is a less expensive and more readily accessible method, but it is still generally considered invasive and may be perceived as time-consuming by clinicians. Therefore, by using ADAS-Cog as a non-invasive, cost-effective, and reliable measure of cognitive impairment, we were able to identify and track changes in cognitive function in AD patients over the time.

In the clinical data set, we found that moderate and high ADAS-Cog groups had significantly lower levels of triglycerides and insulin compared to the low ADAS-Cog group in our phenomics analysis. These findings are consistent with previous studies that have reported a correlation between malnutrition and the progression of AD [1, 33]. Notably, AD can further exacerbate the deterioration of nutritional status due to alterations in appetite, food preference, and eating habits [29]. Interestingly, previous research has shown that medium-chain triglycerides can improve cognition and lipid metabolomics in AD patients in a double-blind, randomized, placebo-controlled crossover trial [72]. Consistent with our findings, a study found that serum triglycerides were decreased in AD compared to normal controls [11]. Furthermore, we observed downregulation of albumin in the moderate group compared to the low group. Low serum albumin has been reported to be associated with cognitive impairment [40].

Our plasma proteomics analyses identified several key features associated with AD progression, including significant downregulation of SKAP1, VPS37A, CALCOCO1, and SF3B4, and significant upregulation of NEFL and GFAP. SKAP1 plays a crucial role in regulating dendritic spine actin dynamics, dendritic spine formation, and maintenance [20], while VPS37A is involved in endosomal sorting [58], synaptic vesicle recycling [25], and autophagy [62]. CALCOCO1 is implicated in the regulation of autophagy [50, 51], and SF3B4 is involved in RNA splicing [12]. Dysregulation of splicing, which is an emerging cause of many neurological disorders, affects various aspects of neurobiology from neurogenesis to synaptic function [66]. Increased NEFL levels are associated with AD and other neurodegenerative disorders [10, 70], while GFAP is an early marker of Aβ pathology in AD [54]. Changes in the expression or function of these proteins may contribute to the development and progression of the disease.

Our analysis of plasma metabolomics revealed significant alterations in amino acids and lipids. Specifically, we found that glutamate, a major excitatory neurotransmitter in the brain, was downregulated in severe AD patients, consistent with previous reports [18, 19, 37, 38]. Previously, some research suggested that glutamate was upregulated in CSF in AD, and excessive activation of the NMDA subtype of glutamate receptors has been implicated in the neurodegenerative processes that lead to AD [43, 68]. However, due to the existence of the blood–brain barrier, the correlation between plasma glutamate and CSF glutamate remains unclear. Additionally, we observed downregulation of HVA, a primary metabolite of the neurotransmitter dopamine, in the high ADAS-Cog group. This finding is important, as changes in dopamine metabolism have been implicated in the development and progression of neurodegenerative disorders such as AD [8]. Furthermore, we identified several significant phospholipids, including PCs and PEs, which have been shown to be associated with AD in previous studies [57, 69]. Lastly, our study found a decrease in L-threonate levels in the high ADAS-Cog group. L-threonate has been reported to increase brain magnesium levels, which may prevent synaptic loss and reverse cognitive deficits in AD [36].

Apart from the metabolites previously discussed, there are several other metabolites that warrant consideration. For example, previous studies have reported a reduction in the concentration of 3-phosphoglycerate and other glycolysis intermediate metabolites in the CSF of individuals with AD [9]. Our analysis revealed significant downregulation of amino acid-related pathways as ADAS scores increased (Supplementary Figure 3A). Specifically, significant downregulation of metabolites related to tyrosine metabolism, glutamate metabolism, branched-chain amino acids (BCAAs) and lysine metabolism was observed. Disruptions in tyrosine metabolism and neurotransmitter synthesis have been reported in various neurological disorders, including AD [48, 71], and changes in tyrosine phosphorylation of proteins involved in synaptic plasticity have also been observed [46]. In addition, several other downregulated metabolites were found to be related to lipid-related pathways, including PCs, PEs, phosphatidylinositols, and secondary bile acid metabolism (Supplementary Figure 3B). Changes in the levels or composition of these lipids have been linked to various neurological disorders, including AD [7, 26, 27, 57, 61].

The potential link between the gut/saliva microbiomes and AD has been increasingly explored in recent research [53, 59]. The gut-brain axis has been implicated in the pathogenesis of AD and alterations in the gut microbiome have been shown to contribute to neurodegeneration and inflammation in the brain [28]. Our study found that *Paraprevotella clara* was significantly increased in the high ADAS-Cog group, while *Phocaeicola dorei* and *Bacteroides fragilis* were significantly decreased. Previous research has shown that *Paraprevotella clara* was significantly increased in patients with attention deficit hyperactivity disorder (ADHD) and ADHD has been associated with an increased risk of AD [52, 67]. Additionally, a lower abundance of Bacteroides has been reported in AD [75], and another study has shown that *Bacteroides fragilis* is present at a lower abundance in patients with cognitive impairment and brain amyloidosis [17]. In MetaPhlAn3 analysis, we also observed that *Roseburia faecis* was downregulated in the high ADAS group (Supplementary Figure 4E). *Roseburia faecis* is part of the commensal bacteria that produce short-chain fatty acids, particularly butyrate, which affects colonic motility, immune maintenance, and anti-inflammatory properties [63]. A decrease in *Roseburia spp.* abundance can affect various metabolic pathways and is associated with several diseases, including irritable bowel syndrome, obesity, type 2 diabetes, nervous system conditions, and allergies [21, 63]. We observed a positive correlation between *Oscillibacter sp. 57_20* and the ADAS-Cog score (Supplementary Table 6) in the MetaPhlAn3 analysis. Additionally, we found that *Anaerostipes hadrus* exhibited significant alterations in AD, as indicated by a *p*-value of 0.057. These findings align with a recent study that identifies *Oscillibacter sp. 57_20* and *Anaerostipes hadrus* as species strongly associated with preclinical AD status [24]. Moreover, emerging evidence suggests that the oral microbiome, including the saliva microbiome, may be associated with AD. Our study found that *Actinomyces israelii* and *Lancefieldella rimae* were upregulated in AD. Poor oral hygiene and periodontal disease, which can alter the oral microbiome, have been linked to an increased risk of AD. Oral bacteria can enter the bloodstream and travel to the brain, triggering neuroinflammation and immune responses linked to AD pathogenesis [53].

Our study incorporated classification models with the goal of precisely categorizing patients into distinct ADAS-Cog groups. These classifiers exhibited exceptional performance, with high accuracy results as illustrated in Fig. 6A. The XGBoost classifier, in particular, was noteworthy for its near-flawless classification of the follow-up cohort, as evidenced in Fig. 6B. However, while the XGBoost regressor demonstrated accurate predictions for ADAS-Cog scores less than 35, it encountered challenges when faced with a patient with a real ADAS-Cog score of 64 (Fig. 6D). This limitation could be linked to the uneven distribution of the training set, especially within the high ADAS-Cog score interval. The scarcity of samples in this range possibly led to the regressor being inadequately trained on high ADAS-Cog score samples. A more balanced and comprehensive sample size could potentially result in a more robust performance from the regressor. To mitigate this limitation, we utilized the K-nearest neighbors (KNN) imputation method to enrich our training data. This approach significantly improved the model's performance on the testing dataset, particularly within the high ADAS-Cog score interval (Fig. 6E).

Our findings suggest that the identified biomarkers collectively indicate evidence for dysregulation of autophagy, a process implicated in various human diseases, including cancer, neurodegenerative diseases, and pathogen infections [44]. Recent studies have demonstrated that several early AD symptoms are paralleled with degeneration of dopamine (DA)-producing neurons, which are involved in regulating cognitive and non-cognitive functions [49]. Notably, researchers found that ventral tegmental area (VTA) DA neurons degenerate early in a validated AD mouse model (Tg2576), potentially due to impaired macroautophagy/autophagy caused by enhanced activity of the ABL/c-Abl kinase [49]. The decrease in HVA, a primary dopamine metabolite, implies a possible decline in dopamine levels. Early autophagic degeneration in dopaminergic neurons of the substantia nigra has been observed in Parkinson’s disease patients [4]. Furthermore, de novo PC synthesis is required for autophagosome membrane formation and maintenance during autophagy [3], while PE abundance positively regulates autophagy [56]. At the proteomics level, autophagy has been reported as a degradative pathway for neurofilament subunit proteins [55], with accumulations of NEFL subunit being pathological hallmarks of amyotrophic lateral sclerosis and contributing to neurofibrillary lesions in AD [32]. Moreover, VPS37A is responsible for recruiting the endosomal sorting complex required for transport (ESCRT) machinery for VPS4-mediated membrane scission and closure of the phagophore [62], while CALCOCO1 has been identified as an ER-phagy receptor, with its depletion causing Golgi expansion and accumulation of Golgi-resident proteins [50, 51].

In conclusion, our findings suggest that dysregulation of neurotransmitters, lipids, and inflammation may be critical drivers of AD pathogenesis. Specifically, we identified several key proteins and metabolites involved in these pathways that could serve as potential biomarkers for AD diagnosis and monitoring (Fig. 7). In addition, our analysis of the gut microbiome revealed significant alterations in the abundance of several bacterial taxa in AD patients, highlighting the potential role of the gut-brain axis in AD pathogenesis (Fig. 7). Overall, our study provides novel insights into the molecular mechanisms underlying AD progression and identifies the potential biomarkers that could aid in early diagnosis and monitoring of AD. Future studies can expand on these findings to investigate the potential therapeutic and diagnostic applications of molecular and microbiome-targeted interventions.

## Star methods

### Lead contact

Further information and requests for resources should be directed to and will be fulfilled by the lead contact, Adil Mardinoglu (adilm@scilifelab.se).

### Clinical trial design and oversight

This study was a phase 2, randomized, double-blinded, placebo-controlled, parallel-group trial with two arms. On day 0 of the study, all enrolled participants were included in the baseline cohort, where comprehensive data were collected prior to the initiation of any treatment. The recruitment of participants took place at the Faculty of Medicine at both Alanya Alaaddin Keykubat University in Antalya, Turkey, and Istanbul Medipol University in Istanbul, Turkey.

The trial adhered strictly to Good Clinical Practice guidelines as well as the principles outlined in the Declaration of Helsinki. The safety of participants and the risk–benefit analysis were supervised by an independent external data-monitoring committee. Approval for this study was granted by the ethics committee of Istanbul Medipol University, Istanbul, Turkey (Approval Date: 22nd January 2020, Decision No: 7).

This study is registered at https://clinicaltrials.gov/, with the Clinical Trial ID: NCT04044131.

### Eligibility criteria of clinical trial participants

Patient enrollment was governed by specific inclusion and exclusion criteria. The inclusion criteria necessitated patients to be of at least 50 years of age and clinically diagnosed with Alzheimer's Disease (AD), as established by the Alzheimer’s Disease Assessment Scale-Cognitive Subscale (ADAS-Cog) with scores greater than or equal to 12. The diagnoses were made in accordance with the Diagnostic and Statistical Manual of Mental Disorders, 5th Edition (DSM-5) criteria.

Conversely, the exclusion criteria disqualified patients with a medical history of stroke, severe brain trauma, or exposure to neurotoxic drugs. Detailed demographic information of the patients is delineated in Supplementary Table 1.

### Proteomics analysis

The quantification of plasma protein levels was performed utilizing Olink panels (Olink Bioscience, Uppsala, Sweden). In brief, each sample was exposed to pairs of DNA-labelled antibodies (proximity probes) through incubation. Upon binding of an antibody pair to its corresponding antigens, the DNA tails create an amplicon via proximity extension, a process that allows quantification through high-throughput, real-time PCR.

We utilized the Olink Explore 1536 platform, which comprises four distinct panels. These panels include the Olink Explore 384 Cardiometabolic Reagent Kit (Panel lot number: B04413), the Olink Explore 384 Inflammation Reagent Kit (Panel lot number: B04411), the Olink Explore 384 Oncology Reagent Kit (Panel lot number: B04412), and the Olink Explore 384 Neurology Reagent Kit (Panel lot number: B04414). Across these four panels, a total of 1472 proteins were targeted using specific antibodies, representing 1463 unique proteins.

For the procedure, 3 μl of the probe solution was combined with 1 μl of the sample, followed by an overnight incubation at 4 °C. A 96 μl extension solution comprising extension enzymes and PCR reagents was subsequently added for the pre-amplification step. The extension products were amalgamated with detection reagents and primers, and loaded onto a chip for qPCR analysis, facilitated by the BioMark HD System (Fluidigm Corporation, South San Francisco, CA).

In an effort to minimize variation both within and between runs, the data were normalized utilizing both internal and interplate controls. Normalized data were presented in arbitrary units (Normalized Protein eXpression, NPX) using a log2 scale and linearized employing the formula 2^NPX. A high NPX corresponded to a high protein concentration. For each assay, the limit of detection was established as three standard deviations above the background, serving as the negative control.

### Untargeted metabolomics analysis

Plasma samples were procured on days 0 and 84, and subjected to untargeted metabolite profiling conducted by Metabolon, located in Durham, NC. The samples were prepared using an automated MicroLab STAR system (Hamilton Company, Reno, NV). For quality assurance, a recovery standard was incorporated prior to the initial extraction stage.

To facilitate the precipitation of proteins and dissociation of small molecules bound to proteins or ensnared within the precipitated protein matrix, and to recover a chemically diverse range of metabolites, methanol was used. This process involved vigorous shaking for 2 min followed by centrifugation.

The extract produced was then apportioned into four fractions. One fraction each was designated for analysis by ultraperformance liquid chromatography-tandem mass spectroscopy (UPLC-MS/MS) with positive ion-mode electrospray ionization, and UPLC-MS/MS with negative ion-mode electrospray ionization. A third fraction was analyzed using gas chromatography-mass spectrometry, while the fourth fraction was preserved as a backup.

### Metagenomics data analysis

Fresh specimens of stool and saliva were procured and preserved using DNA/RNA Shield Fecal Collection tubes and DNA/RNA Shield Collection Tube respectively, both provided by Zymo Research, Irvine, CA. DNA extractions from the fecal samples were carried out employing the QIAamp PowerFecal Pro DNA Kit (Qiagen, Hilden, Germany), while the saliva samples were processed using the QIAamp DNA Microbiome Kit (Qiagen, Hilden, Germany). All protocol procedures adhered strictly to the manufacturer’s guidelines.

Quantification of the extracted DNA was executed fluorometrically with the Qubit 3.0 Fluorometer (Thermo Fisher Scientific, United States), utilizing the Qubit™ dsDNA HS Assay Kit. DNA purity was ascertained through the 260/280 and 260/230 ratios measured on the NanoDrop 1000 (Thermo Fisher Scientific, United States). The SMARTer Thruplex DNA-Seq (Takara Bio) was employed for library preparation, following the low input, 350 bp option.

Sequencing of samples was performed on NovaSeq6000 (NovaSeq Control Software 1.7.0/RTA v3.4.4) with a 151nt (Read1)-10nt(Index1)-10nt(Index2)-151nt(Read2) configuration, using the “NovaSeqXp” workflow on an “S4” mode flow cell. The conversion from Bcl to FastQ was conducted utilizing bcl2fastq_v2.20.0.422 from the CASAVA software suite, with the Sanger/phred33/Illumina 1.8 + quality scale.

Taxonomic profiles for each sample were derived from the raw paired-end metagenomics data through the use of MetaPhlAn4 [13]. The Kolmogorov–Smirnov test was applied to the abundance data to identify any differences in species distribution between different subject groups. The Mann–Whitney U test was utilized to determine if the distribution of one group stochastically exceeded the other. In addition, a correlation analysis was conducted between saliva and gut metagenomics data, filtering species with a prevalence greater than 20% within the cohort. All statistical analyses were conducted using the SciPy package in Python 3.9.13.

### Statistical analysis

To evaluate the independence of our division into ADAS-Cog groups with respect to gender and smoking habits, Chi-squared tests were employed. Differences in age and BMI distributions among the ADAS-Cog groups were examined using Kolmogorov–Smirnov tests. The significance of results from proteomics and metabolomics analyses was established through the Kruskal–Wallis one-way analysis of variance.

All statistical analyses were conducted with SciPy 1.10.1, and the false discovery rate was corrected utilizing the Benjamini–Hochberg method in statsmodels 0.14.0. For visualizing graphs, matplotlib 3.7.0 and seaborn v0.12 were used. UMAP visualization was applied to clinical data, utilizing two components and 25 neighbors, with a random seed of 0.

All of the above analyses were performed using Python 3.9.13.

## Supplementary Information

Supplementary Material 1: Supplementary Figure 1. Correlation analysis between significant clinical parameters and the top 40 plasma proteins. Supplementary Figure 2. Correlation plots depicting the relationship between ADAS-Cog scores and the protein levels of SKAP1, VPS37A, CALCOCO1, and SF3B4, respectively. Supplementary Figure 3. Heat map displaying all the plasma metabolites that are significantly altered between different patient groups. (A) Amino acids and their derivatives. (B) Lipids. Supplementary Figure 4. Significant differences in gut and saliva species abundance between different patient groups. (A) Gut species that significantly differ between the high and moderate ADAS-Cog groups. (B) Gut species that significantly differ between the moderate and low ADAS-Cog groups. (C) Saliva species that significantly differ between the high and moderate ADAS-Cog groups. (D) Saliva species that significantly differ between the moderate and low ADAS-Cog groups. (E) Gut species that significantly differ between the high and moderate ADAS-Cog groups with MetaPhlAn3. (F) Saliva species that significantly differ between the high and moderate ADAS-Cog groups with MetaPhlAn3. Supplementary Figure 5. Correlation analyses between different omics data. (A) Correlation between top 10 plasma proteins and top 40 plasma metabolites. (B) Correlation between top 10 plasma proteins and top 20 gut microbiomes. (C) Correlation between top 10 plasma proteins and top 20 saliva microbiomes. (D) Correlation between top 10 plasma metabolites and top 20 gut microbiomes. (E) Correlation between top 10 plasma metabolites and top 20 saliva microbiomes. (F) Correlation between top 20 gut and saliva microbiomes. Supplementary Figure 6. (A) Feature importance as identified on the testing (day 84) dataset using the XGBoost algorithm. (B) Unsupervised learning using MOFA+ to present feature variance. (C) Feature importance analysis of clinical parameters among the top 5 factors. (D) Feature importance analysis of plasma protein parameters among the top 5 factors. (E) Feature importance analysis of plasma metabolite parameters among the top 5 factors.

Supplementary Material 2.

Supplementary Material 3.

Supplementary Material 4.

Supplementary Material 5.

Supplementary Material 6.

Supplementary Material 7.

Supplementary Material 8.

## Data Availability

The data and code produced during the course of this research can be accessed from our GitHub repository at https://github.com/lingqime/AD-baseline. Please note that for access to the raw clinical data, interested parties are requested to directly get in touch with the designated lead contact.
