# Supplementary figures and images for: Multi-omics analysis reveals the key factors involved in the severity of the Alzheimer’s disease
Source: Alzheimers Res Ther. 2024 Oct 2;16:213. doi: 10.1186/s13195-024-01578-6 (PMC11448018; doi:10.1186/s13195-024-01578-6)

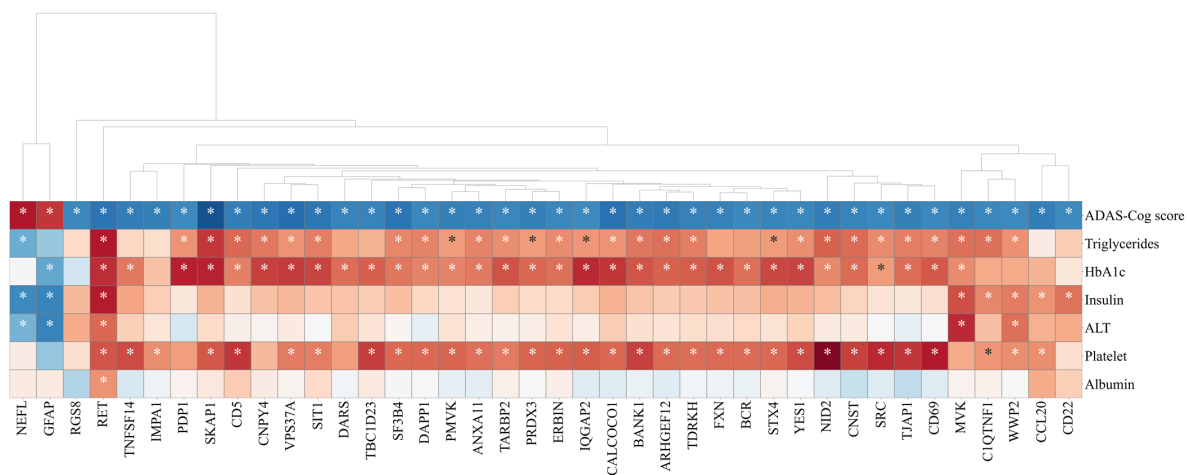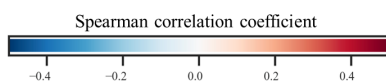

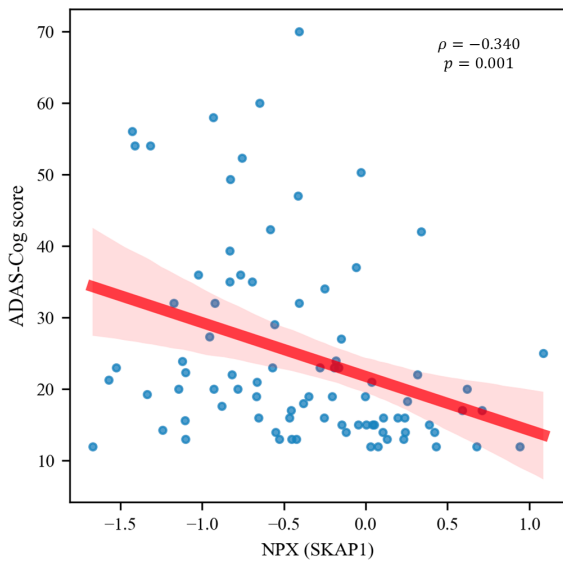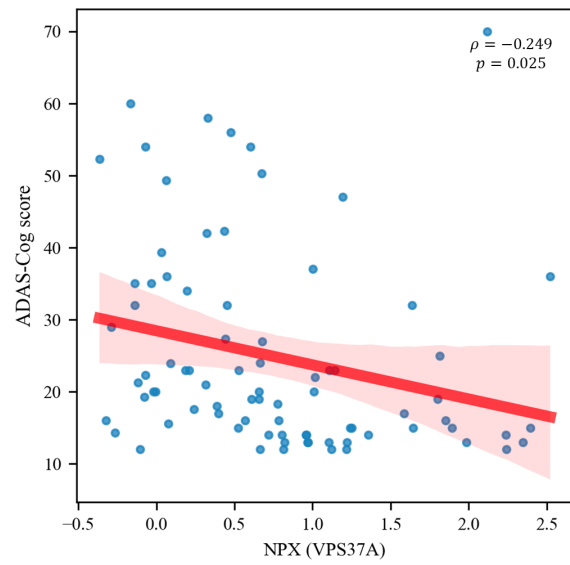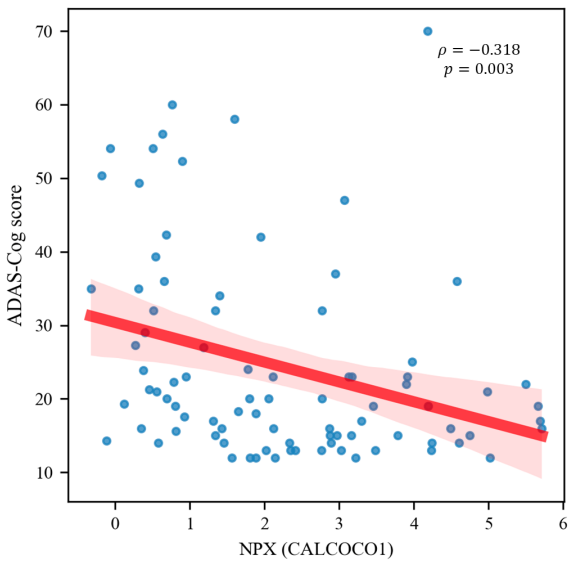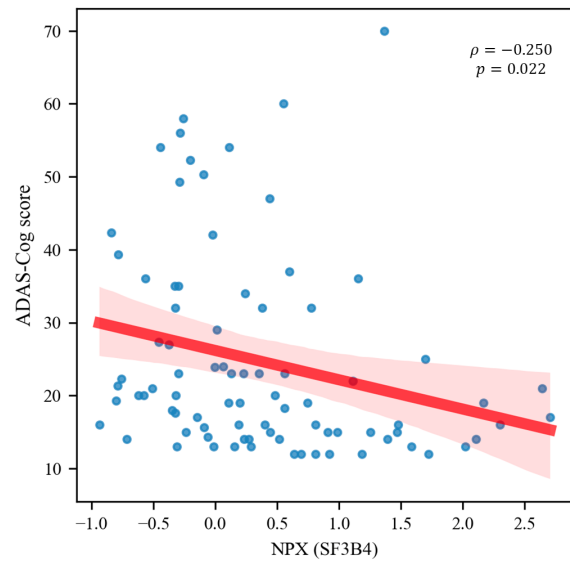

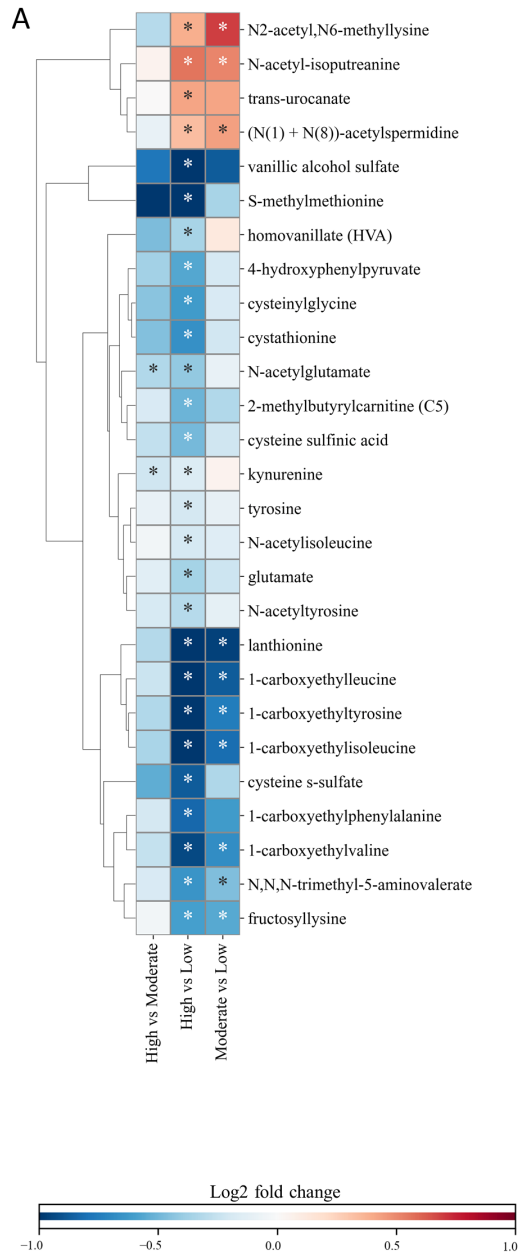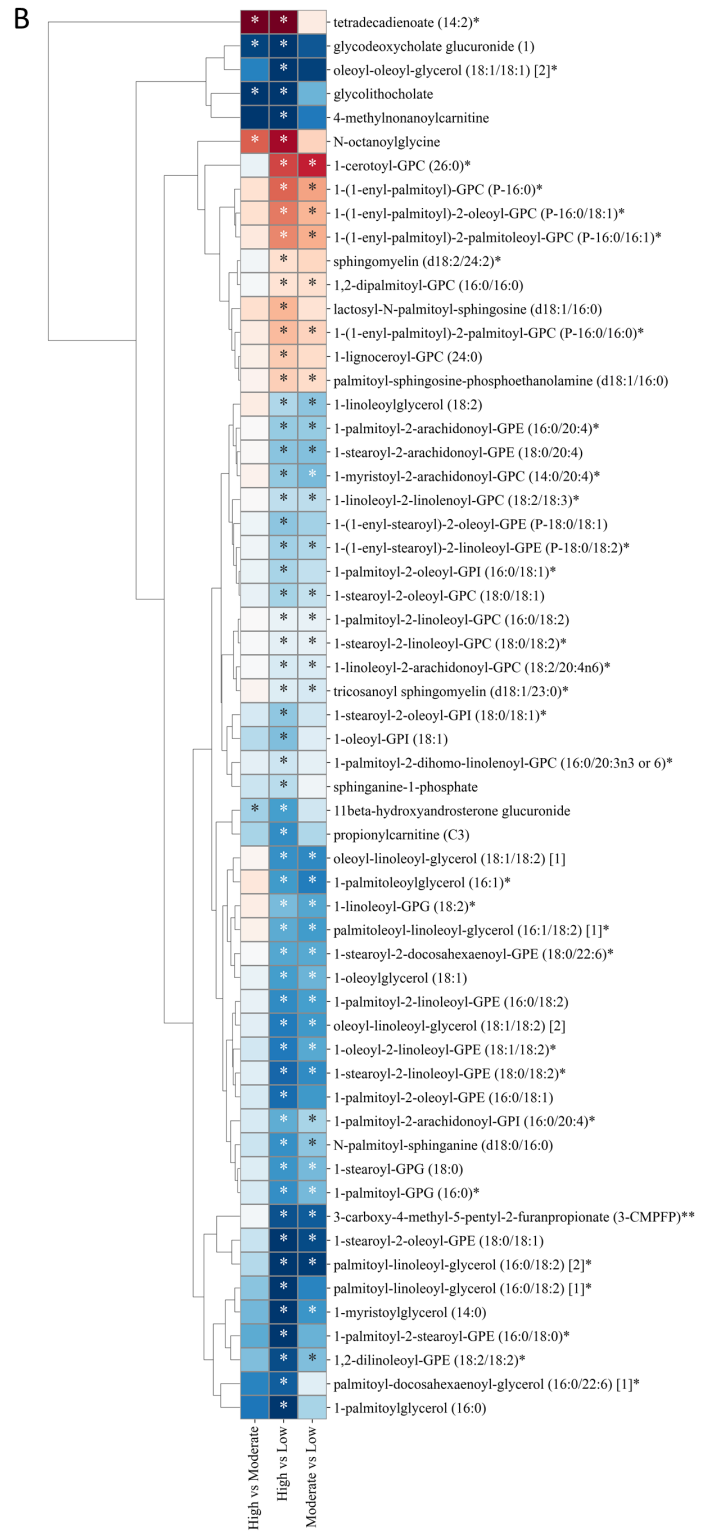

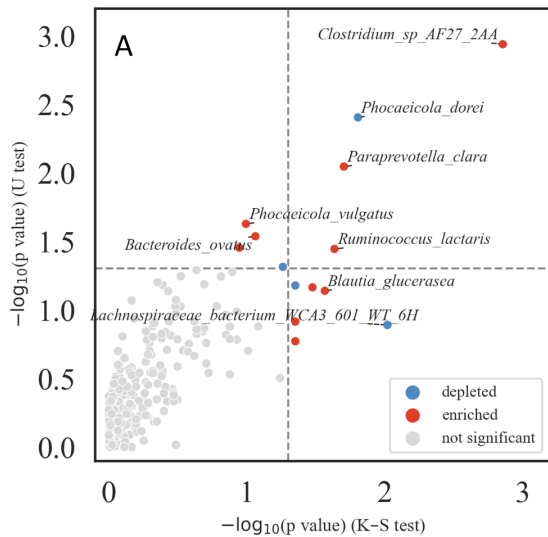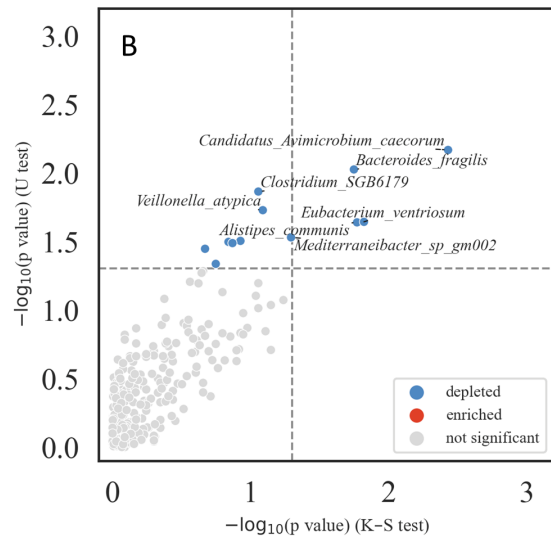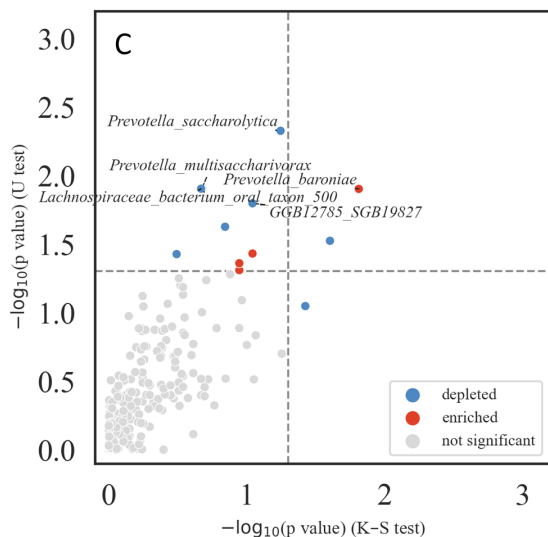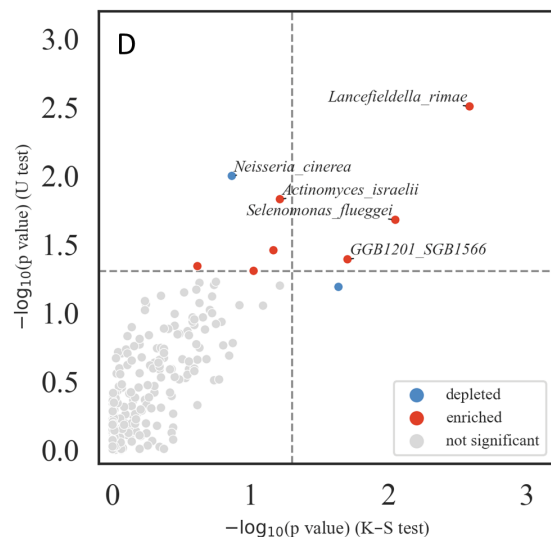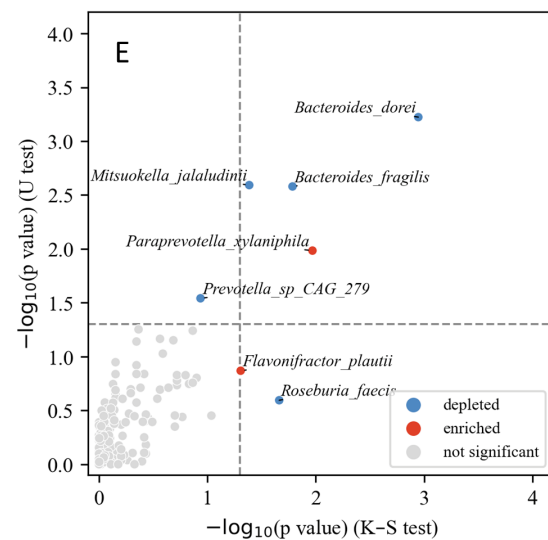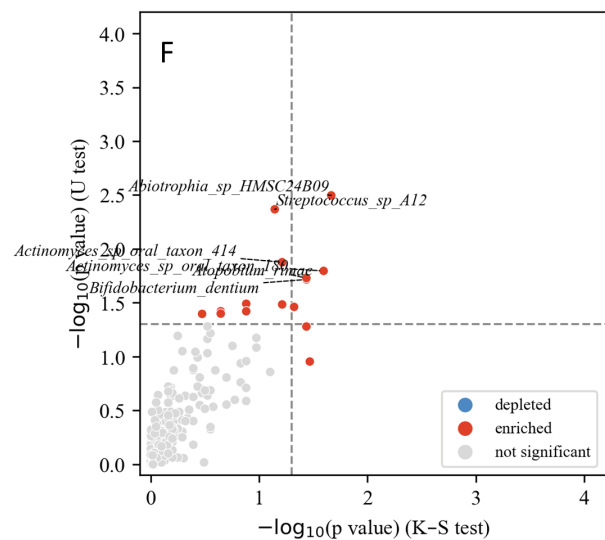

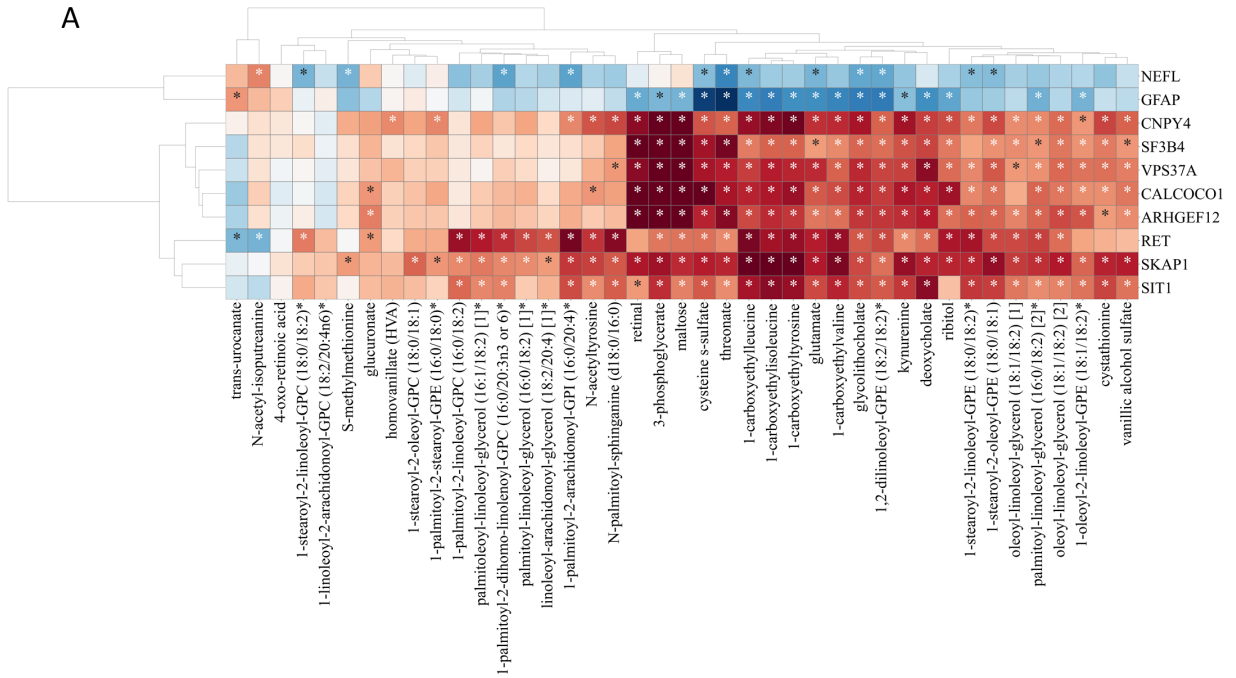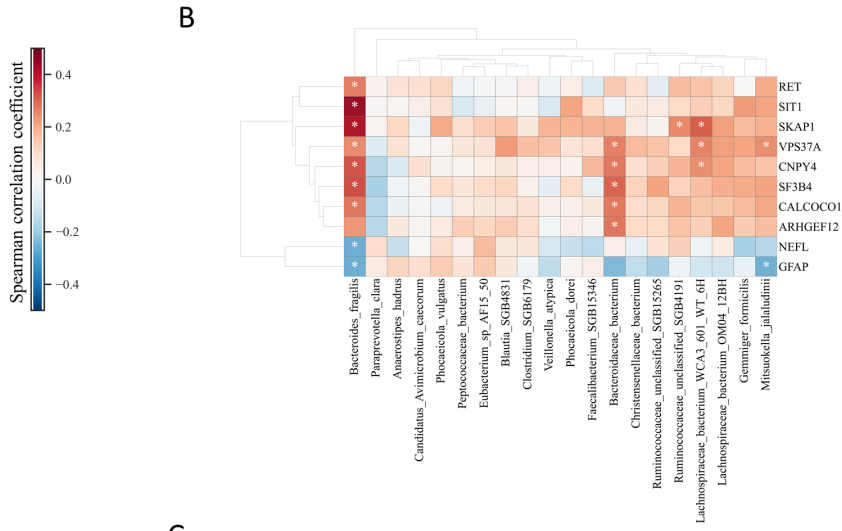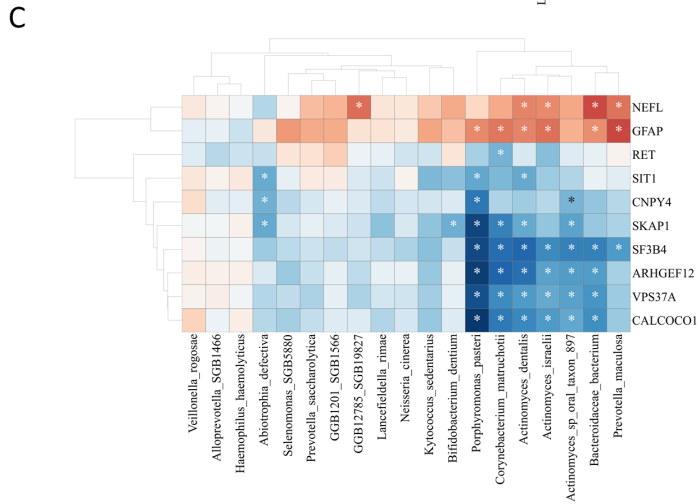

D

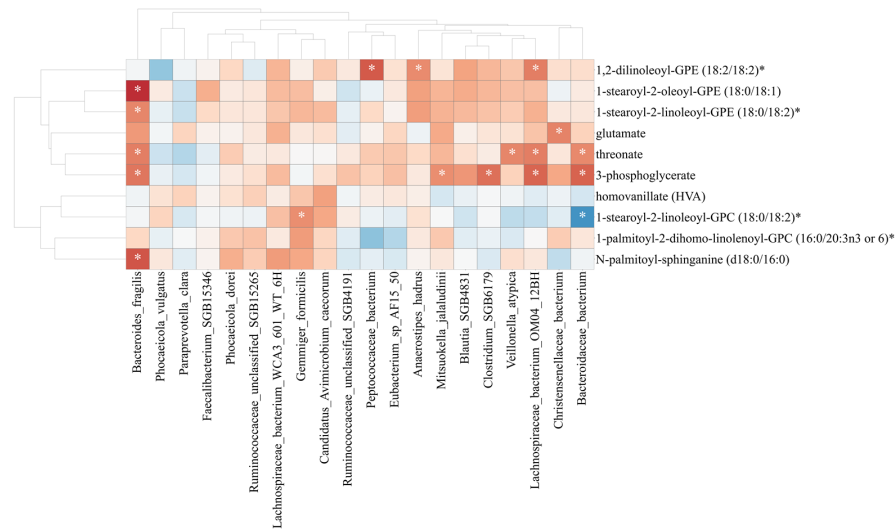

E

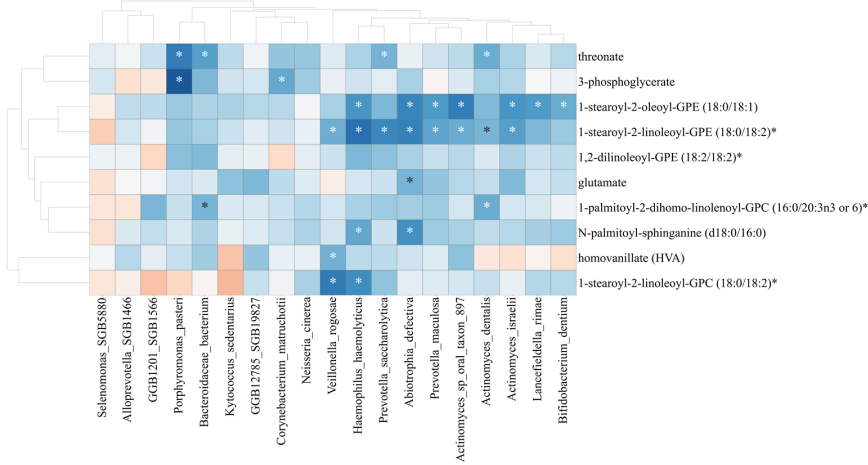

F

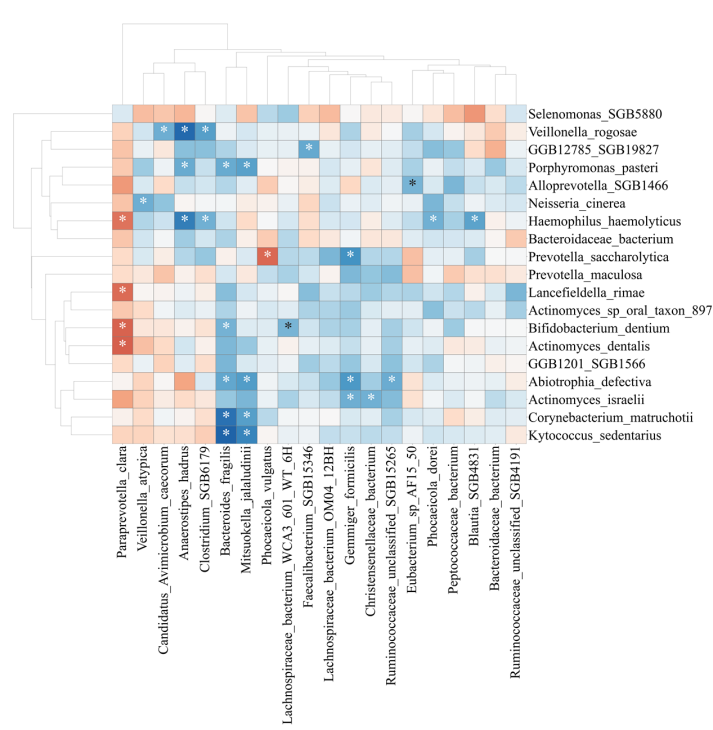

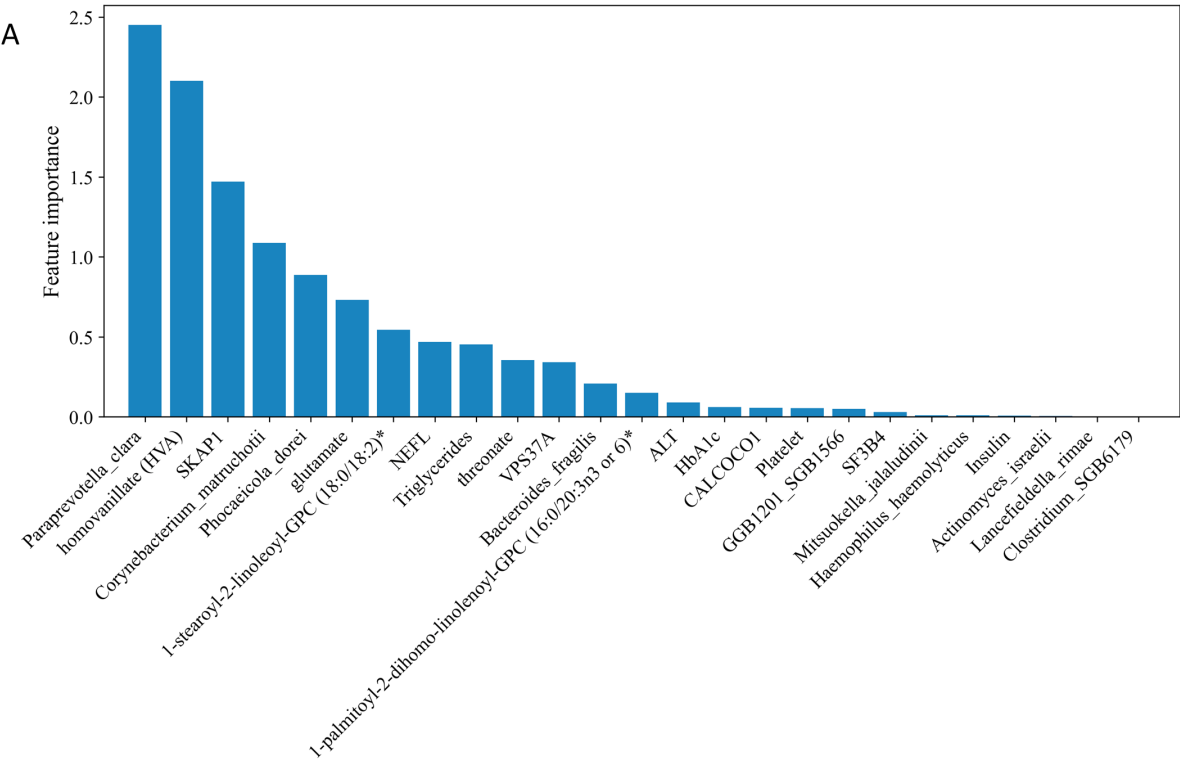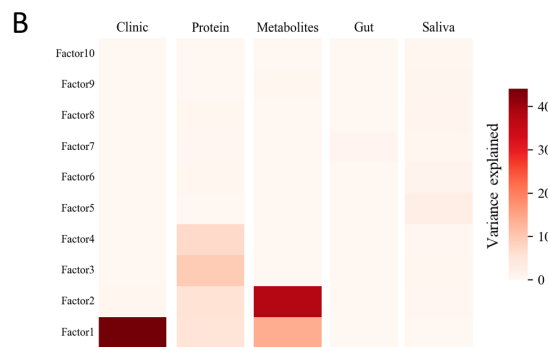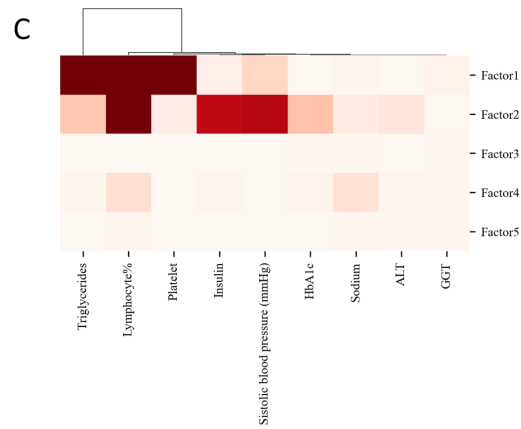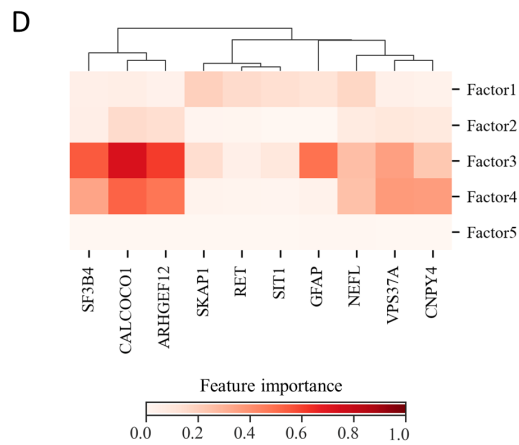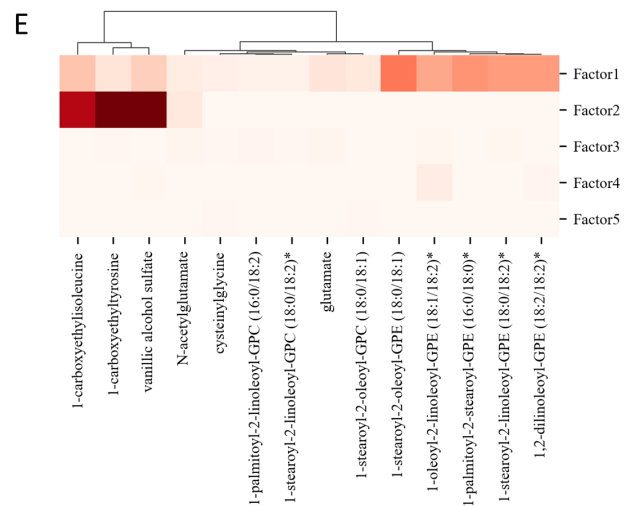

Supplement: Supplementary file 1 — Supplementary Material 1: Supplementary Figure 1. Correlation analysis between significant clinical parameters and the top 40 plasma proteins. Supplementary Figure 2. Correlation plots depicting the relationship between ADAS-Cog scores and the protein levels of SKAP1, VPS37A, CALCOCO1, and SF3B4, respectively. Supplementary Figure 3. Heat map displaying all the plasma metabolites that are significantly altered between different patient groups. (A) Amino acids and their derivatives. (B) Lipids. Supplementary Figure 4. Significant differences in gut and saliva species abundance between different patient groups. (A) Gut species that significantly differ between the high and moderate ADAS-Cog groups. (B) Gut species that significantly differ between the moderate and low ADAS-Cog groups. (C) Saliva species that significantly differ between the high and moderate ADAS-Cog groups. (D) Saliva species that significantly differ between the moderate and low ADAS-Cog groups. (E) Gut species that significantly differ between the high and moderate ADAS-Cog groups with MetaPhlAn3. (F) Saliva species that significantly differ between the high and moderate ADAS-Cog groups with MetaPhlAn3. Supplementary Figure 5. Correlation analyses between different omics data. (A) Correlation between top 10 plasma proteins and top 40 plasma metabolites. (B) Correlation between top 10 plasma proteins and top 20 gut microbiomes. (C) Correlation between top 10 plasma proteins and top 20 saliva microbiomes. (D) Correlation between top 10 plasma metabolites and top 20 gut microbiomes. (E) Correlation between top 10 plasma metabolites and top 20 saliva microbiomes. (F) Correlation between top 20 gut and saliva microbiomes. Supplementary Figure 6. (A) Feature importance as identified on the testing (day 84) dataset using the XGBoost algorithm. (B) Unsupervised learning using MOFA+ to present feature variance. (C) Feature importance analysis of clinical parameters among the top 5 factors. (D) Feature impor [file 13195_2024_1578_MOESM1_ESM.pdf]
